# Effectiveness of dolutegravir-based regimens compared to raltegravir-, elvitegravir-, bictegravir, and darunavir-based regimens among older adults with HIV in the Veterans Aging Cohort Study (VACS)

**DOI:** 10.1186/s12981-024-00681-w

**Published:** 2024-12-21

**Authors:** Lei Yan, Cassidy E. Henegar, Vincent C. Marconi, Kirsha S. Gordon, Charles Hicks, Vani Vannappagari, Amy C. Justice, Mihaela Aslan

**Affiliations:** 1 Veterans Affairs (VA) Connecticut Healthcare System Cooperative Studies Program Clinical Epidemiology Research Center (CSP-CERC), 950 Campbell Avenue, West Haven, CT 06516-2770 USA; 2 Yale University School of Public Health, New Haven, CT USA; 3 ViiV Healthcare, Durham, NC USA; 4 Emory University School of Medicine and Rollins School of Public Health, Atlanta, GA USA; 5 Atlanta VA Medical Center, Atlanta, GA USA; 6 Yale University School of Medicine, New Haven, CT USA

**Keywords:** HIV, Antiretroviral therapy, Effectiveness, Discontinuation

## Abstract

**Background:**

Real-world data on treatment patterns and clinical outcomes for newer drugs, including integrase strand transfer inhibitors, among older people with human immunodeficiency virus (PWH) are limited.

**Methods:**

This cohort study included PWH enrolled in the Veterans Aging Cohort Study (VACS) who were prescribed a standard 3-drug antiretroviral therapy (ART) regimen containing dolutegravir (DTG), bictegravir (BIC), cobicistat boosted elvitegravir (EVG), raltegravir (RAL), or darunavir/ritonavir (DRV) plus 2 nucleoside reverse transcriptase inhibitors between January 1, 2014, and March 31, 2020, and who were ≥50 years at regimen initiation. The association between regimen and virologic effectiveness or discontinuation was assessed using logistic regression models with inverse probability of treatment weights. Pairwise comparisons were made between DTG-based regimen and each of the other 3-drug regimens, stratified by ART experience.

**Results:**

Among 15,702 PWH (across treatment groups, median age 58–62 years; 94–98% male; 5–11% Hispanic; 44–60% Black; 29–42% White), 5,800 received DTG-based regimens, 2,081 BIC-based regimens, 4,159 EVG-based regimens, 1,607 RAL-based regimens, and 2,055 received DRV-based regimens. Among ART-naïve PWH, there were no statistical differences in the odds of virologic suppression, and 6- and 12-month discontinuations were higher in those on DRV. Among ART-experienced PWH, compared to DTG, those on RAL and DRV were less likely to be suppressed at 6 months (RAL vs DTG: aOR 0.64, 95% CI 0.51–0.81; DRV vs DTG: aOR 0.63, 95% CI 0.51–0.76) and those on EVG and DRV were less likely suppressed at 12 months (EVG vs DTG: aOR 0.82, 95% CI 0.68–0.99; DRV vs DTG: aOR 0.64, 95% CI 0.52–0.80). Those on DRV were more likely to have virologic failure within 12 months (aOR 1.96, 95% CI 1.30–2.97). Six- and 12-month discontinuations were higher in those on RAL and DRV, but less likely for BIC-based regimens.

**Conclusions:**

DTG-based regimens demonstrated higher levels of effectiveness and durability compared to DRV- or RAL-based regimens and had similar treatment responses as BIC- and EVG-based regimens among ART-experienced older PWH.

**Supplementary Information:**

The online version contains supplementary material available at 10.1186/s12981-024-00681-w.

## Background

Substantial progress has been made with respect to ART in terms of lower pill burden and reduced dosing frequency, high levels of tolerability with fewer side effects, and higher barriers to resistance when compared to older treatment options [1, 2]. However, there are limited data on ART patterns and response among older PWH, especially for newer drug classes of antiretrovirals, including integrase strand transfer inhibitors (INSTIs) [3, 4]. Immune function naturally deteriorates with age [5, 6]. For PWH, this deterioration exacerbates the underlying immune dysfunction that occurs with HIV infection, resulting in a higher risk and earlier onset of many age-related comorbidities. Exposure to ART may also contribute to the development of age-related conditions [7]. Pharmacodynamic and pharmacokinetic parameters may also differ by age, potentially leading to more adverse effects from ARVs among older PWH [8]. Thus, choosing appropriate and well-tolerated ART regimens is crucial for addressing the challenges posed by an aging population with HIV [9–11].

The Veterans Aging Cohort Study (VACS) is a prospective, observational cohort study of military veterans in the United States with and without HIV infection [12, 13]. The VACS is embedded within the Veterans Health Administration (VHA) which represents the largest integrated healthcare system and the largest single provider of HIV care in the United States with over 76,000 veterans aged 50 years or older. We sought to compare virologic effectiveness, regimen discontinuation, and immunologic response with common first-line 3-drug regimens among older PWH enrolled in a real-world setting.

## Methods

### Setting and data sources

We used data from VACS to describe the overall clinical characteristics and response to modern 3-drug antiretroviral regimens among PWH ≥50 years old at the time of regimen initiation. Detailed descriptions of VACS have been provided elsewhere [12, 13]. In brief, VACS is a longitudinal, prospective cohort that includes individuals with and without HIV (matched 1:2 on age, race/ethnicity, sex, and site-of-care) identified within the VA electronic health records (EHR) system, which includes demographic characteristics, outpatient diagnoses (recorded using International Classification of Diseases, Ninth Revision [ICD-9] and Tenth Revision [ICD-10] codes), laboratory results, and dispensed medications.

### Eligibility criteria and study population

Eligible individuals were PWH prescribed a 3-drug ART regimen containing a single core agent of dolutegravir (DTG), bictegravir (BIC), cobicistat boosted elvitegravir (EVG), raltegravir (RAL), or ritonavir boosted darunavir (DRV) with 2 nucleoside reverse transcriptase inhibitors (Supplemental Tables 1 and 2) between January 1, 2014 and March 31, 2020, who were at least 50 years old at regimen initiation. Exposure was defined by the core agent prescribed (DTG, BIC, EVG, RAL, and DRV). All analyses were stratified by treatment status (ART-naïve and ART-experienced at regimen initiation). If multiple eligible regimens were prescribed during this study window, follow up was restricted to the first regimen. Those who were suppressed but lacked information on previous regimens were excluded. Follow-up began at regimen initiation and ended at the earliest event of regimen discontinuation (defined as a change to the core agent, change in total number of drugs, or a prolonged ART interruption which was 2 times the days of supply of the previous prescription), death (all-cause), loss to follow-up (defined as lack of clinic visits within 6 months), or end of the clinical data (March 31, 2021).

### Baseline characteristics

The baseline date was the first documented prescription for an eligible ART regimen. Baseline characteristics were assessed, including demographic variables, health factors, metabolic and lipid profiles, comorbidities, baseline CD4 + T-cell count, baseline viral load (VL), and number of non-ART medications. For ART-experienced PWH switching to a study-eligible regimen, the number of ART regimens previously used and time since ART initiation were reported. VACS index 2.0 was used to measure disease severity [14]. VACS index 2.0 is a physiologic score that predicts the risk of all-cause mortality based on age, HIV biomarkers (CD4 and VL), and non-HIV biomarkers such as hemoglobin, hepatitis C, fibrosis-4 (to assess liver function), estimated glomerular filtration rate (to assess renal function), albumin, BMI and white blood cell count, with higher scores indicating a greater risk of mortality [14]. Baseline laboratory levels were ascertained within 6 months prior to regimen initiation.

### Outcomes

The primary objective of this analysis was to assess and compare virologic suppression (VS; VL < 50 copies/mL) 6- (±3 months) and 12-(±3 months) months after regimen initiation (Supplemental Fig. 1). Additional virologic outcomes included low-level viremia (VL ≥ 50 copies/mL and < 200 copies/mL) 6 (±3) months and 12(±3 months) after initiation, virologic non-response among ART-naïve and ART-experienced non-suppressed at baseline (2 consecutive VL ≥200 copies/mL after at least 24 weeks of treatment with regimen) and virologic failure (VL ≥ 200 copies/mL in two consecutive measurements within 12 months of regimen initiation or one VL ≥ 200 copies/mL in one measurement within 12 months of regimen initiation followed by regimen discontinuation within the subsequent 4 months, evaluated post-baseline for ART-experienced suppressed and post-suppression for ART-naïve and ART-experienced viremic at baseline).

Immunologic response was assessed as change in CD4 count (cells/uL) from baseline to the end of 6 months as a continuous measure. Additionally, regimen discontinuation, by 6- and 12-months post-baseline regimen initiation, was evaluated. Change in VACS index 2.0 from baseline to the end of 6 months was also estimated to evaluate change in disease severity.

### Statistical analyses

Baseline characteristics were summarized using counts and proportions for categorical variables and median and interquartile range (IQR) for continuous variables. The differences in each variable across regimen groups was assessed using a Chi-squared test for categorical variables and an analysis of variance (ANOVA) test for numerical variables, with the *p*-value reported. All outcomes were estimated using multivariate models with inverse probability of treatment weighting (IPTW) to adjust for confounding by treatment assignment [15–17]. IPTW weights were constructed using propensity scores. We determined the propensity score of each patient receiving one treatment versus another by constructing a distinct logistic regression model, adjusted for age, sex, race and ethnicity, region, smoking status, self-reported and ICD-9/10 based alcohol use disorder, ICD-9/10 based drug use and dependence, ICD-9/10 based homelessness, baseline low-density lipoprotein, baseline CD4 count, baseline VL, and baseline VACS index 2.0. Years on ART regimen was also included for ART-experienced individuals. Stabilized weights were calculated and trimmed at 99th percentile to remove extreme values [18]. Variable balance was assessed by comparing standardized mean differences before and after applying the treatment weights [19]. Treatment weights were then used to fit weighted logistic or linear regression models to estimate the treatment effects on binary and continuous outcomes, respectively. For the outcome models, we adjusted for the same set of variables used in the weighting model.

DTG-based 3-drug regimen served as the referent regimen. Assessments of outcomes at time points (6- and 12-month evaluations) were limited to those who had complete data on baseline and outcome variables for the specified timepoint or time period. Missing data and deaths are summarized in Supplemental Tables 3 and 4.

ART-experienced individuals were stratified by age group (50–64 years and ≥65 years) and hepatitis C virus (active HCV and no active HCV) to estimate effectiveness of DTG-based 3-drug regimens versus the other 3-drug regimens in prespecified population groups. The subgroup analysis was not performed for ART-naïve individuals due to small sample size.

### Sensitivity analyses

For missing values in baseline variables used in the weighting model, we created a missingness indicator for each variable and incorporated it into weight estimation (whereas in the primary approach, we used complete cases). Second, we applied inverse probability of censoring weight to account for those whose outcomes were missing due to unavailability of labs at 6 or 12 months [20, 21] (whereas in the primary approach, we removed those who had missing outcomes). Last, we restricted the DTG versus BIC comparison to the period following February 7, 2018, the date on which BIC was approved (initiation year of regimens reported in Supplemental Table 5).

Analyses and data visualizations were performed with R 4.2.0 (R Project for Statistical Computing). The study was approved by the VA Connecticut Healthcare System institutional review board, which granted a waiver of informed consent because of the retrospective nature of the study. The study followed the Strengthening the Reporting of Observational Studies in Epidemiology (STROBE) reporting guidance.

## Results

### Baseline characteristics

**Fig. 1:**
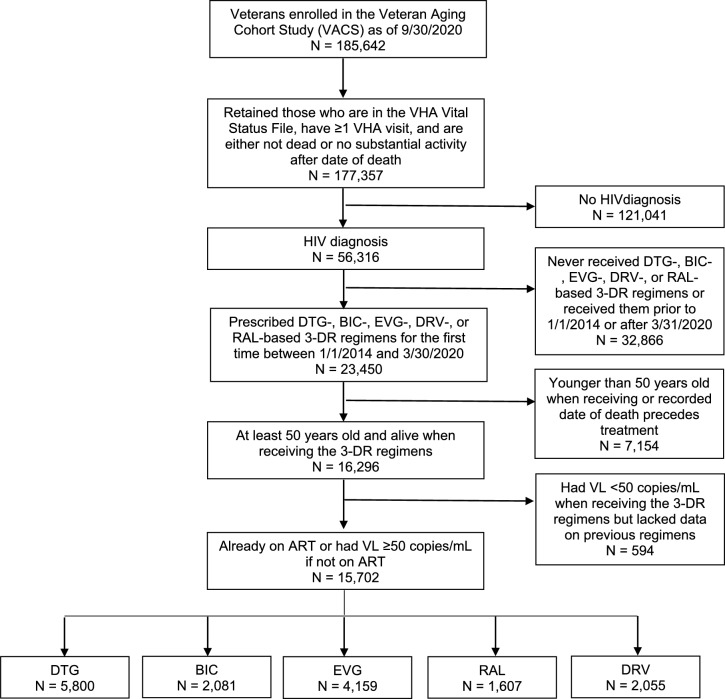
Study flowchart. *VHA* Veterans Health Administration, *DTG* dolutegravir, *BIC* bictegravir, *EVG* elvitegravir, *RAL* raltegravir, *DRV* darunavir, *DR* drug, *ART* antiretroviral therapy, *VL* viral load

A total of 15,702 individuals were eligible for inclusion in our study (Fig. 1). Among 1,895 (12.1% of the cohort) ART-naïve individuals, 703 (37.1%) received DTG, 316 (16.7%) received BIC, 579 (30.6%) received EVG, 121 (6.4%) received RAL, and 176 (9.3%) received DRV-based regimens (Table 1A). Among 13,807 (87.9% of the cohort) ART-experienced individuals, 5,097 (36.9%) received DTG, 1,765 (12.8%) received BIC, 3,580 (25.9%) received EVG, 1,486 (10.8%) received RAL, and 1,879 (13.6%) received DRV-based regimens (Table 1B).

**Table 1:** Baseline demographic and clinical characteristics of the study cohort

	Total (N = 1895)	DTG (N = 703, 37.1%)	BIC (N = 316, 16.7%)	EVG (N = 579, 30.6%)	RAL (N = 121, 6.4%)	DRV (N = 176, 9.3%)	*P*-value^a^
*A. ART-naïve*							
Age, median (IQR)	59 (55–64)	59 (55–64)	60 (56–65)	58 (54–63)	59 (55–65)	59 (54–65)	<0.001
Age (≥65), n (%)	423 (22)	157 (22)	86 (27)	113 (20)	24 (20)	43 (24)	0.096
Male, n (%)	1809 (95)	663 (94)	306 (97)	554 (96)	117 (97)	169 (96)	0.387
Race and Ethnicity, n (%)							0.271
Hispanic	116 (6)	34 (5)	20 (6)	33 (6)	13 (11)	16 (9)	
Black, nonhispanic	1005 (53)	382 (54)	167 (53)	303 (52)	56 (46)	97 (55)	
White, nonhispanic	671 (35)	243 (35)	113 (36)	215 (37)	47 (39)	53 (30)	
Other/missing	103 (5)	44 (6)	16 (5)	28 (5)	5 (4)	10 (6)	
Region							0.040
Midwest	277 (15)	107 (15)	54 (17)	73 (13)	19 (16)	24 (14)	
Northeast	364 (19)	130 (18)	59 (19)	107 (18)	31 (26)	37 (21)	
Southeast	742 (39)	289 (41)	111 (35)	244 (42)	47 (39)	51 (29)	
Southwest	220 (12)	79 (11)	45 (14)	63 (11)	8 (7)	25 (14)	
West	292 (15)	98 (14)	47 (15)	92 (16)	16 (13)	39 (22)	
Smoking, n (%)							0.001
Current	957 (51)	371 (53)	134 (42)	286 (49)	75 (62)	91 (52)	
Past	586 (31)	211 (30)	127 (40)	176 (30)	22 (18)	50 (28)	
Never/Unknown	352 (19)	121 (17)	55 (17)	117 (20)	24 (20)	35 (20)	
Alcohol use disorder (recent 12 m), n (%)	458 (24)	168 (24)	77 (24)	149 (26)	28 (23)	36 (20)	0.698
Drug use and dependence (recent 12 m), n (%)	313 (17)	116 (17)	50 (16)	89 (15)	26 (21)	32 (18)	0.528
Homeless (recent 12 m), n (%)	251 (13)	92 (13)	37 (12)	78 (13)	16 (13)	28 (16)	0.777
Statin, n (%)	446 (24)	200 (28)	96 (30)	101 (17)	27 (22)	22 (12)	<0.001
Total cholesterol (mg/dl), median (IQR)	161 (134–188)	161 (132–187)	157 (136–182)	164 (139–189)	155 (121–175)	161 (135–193)	0.055
High-density lipoprotein (mg/dl), median (IQR)	39 (30–49)	39 (30–49)	39 (31–49)	39 (32–50)	37 (27–47)	36 (30–46)	0.373
Low-density lipoprotein (mg/dl), median (IQR)	94 (72–116)	93 (71–114)	93 (71–116)	97 (76–118)	85 (63–112)	94 (76–118)	0.055
Triglyceride (mg/dl), median (IQR)	115 (84–168)	113 (85–169)	113 (79–162)	118 (85–166)	120 (86–188)	118 (92–160)	0.802
Hemoglobin (g/dl), median (IQR)	13 (12–15)	13 (12–15)	13 (12–14)	14 (12–15)	14 (12–15)	13 (11–14)	<0.001
A1C (%), median (IQR)	5.7 (5.4–6.2)	5.7 (5.4–6.2)	5.8 (5.3–6.2)	5.7 (5.3–6.1)	5.8 (5.5–6.4)	5.7 (5.4–6.2)	0.013
Estimated glomerular filtration rate (ml/min/1.73 m^2^), median (IQR)	76 (60–94)	73 (60–94)	76 (60–94)	77 (60–95)	82 (60–98)	79 (60–94)	0.384
Obesity (BMI ≥ 30 kg/m^2^), n (%)	433 (23)	171 (26)	73 (25)	131 (24)	25 (21)	33 (21)	0.677
Diabetes (ever), n (%)	404 (21)	169 (24)	77 (24)	90 (16)	39 (32)	29 (16)	<0.001
Diabetes (type 2), n (%)	402 (21)	168 (24)	77 (24)	89 (15)	39 (32)	29 (16)	<0.001
Hypertension, controlled, with Rx, n (%)	624 (33)	250 (36)	106 (34)	186 (32)	34 (28)	48 (27)	0.181
Hypertension, uncontrolled, n (%)	528 (28)	170 (24)	87 (28)	187 (32)	26 (21)	58 (33)	0.004
Cardiovascular disease (ever), n (%)	208 (11)	91 (13)	39 (12)	50 (9)	15 (12)	13 (7)	0.056
Hepatitis B virus infection (ever), n (%)	46 (2)	17 (2)	6 (2)	15 (3)	3 (2)	5 (3)	0.967
Hepatitis C virus infection (ever), n (%)	252 (13)	98 (14)	36 (11)	71 (12)	25 (21)	22 (12)	0.109
Major depression (ever), n (%)	428 (23)	183 (26)	68 (22)	115 (20)	26 (21)	36 (20)	0.092
Comorbidities, n (%)							<0.001
0	1151 (61)	385 (55)	269 (85)	356 (61)	51 (42)	90 (51)	
1	349 (18)	121 (17)	20 (6)	126 (22)	33 (27)	49 (28)	
≥2	395 (21)	197 (28)	27 (9)	97 (17)	37 (31)	37 (21)	
CD4 (cells/uL), median (IQR)	318 (141–520)	334 (170–534)	316 (126–520)	358 (179–574)	294 (124–461)	159 (86–344)	<0.001
Viral load (copies/mL), median (IQR)	46,850 (9127–156,248)	46,800 (9885–146,500)	55,624 (6304–215000)	35,977 (9638–134,907)	38,810 (4420–142823)	70,800 (14,058–300500)	0.156
VACS index 2.0, median (IQR)	66 (57–77)	66 (57–78)	66 (58–78)	64 (55–74)	70 (60–80)	71 (61–84)	<0.001
Non-ARV co-medications (recent 12 m), median (IQR)	7 (3–12)	8 (3–14)	7 (3–13)	6 (2–11)	7 (4–12)	5 (1–10)	<0.001

	Total (N = 13,807)	DTG (N = 5097, 36.9%)	BIC (N = 1765, 12.8%)	EVG (N = 3580, 25.9%)	RAL (N = 1486, 10.8%)	DRV (N = 1879, 13.6%)	*P*-value^a^
*B. ART-experienced*							
Age, median (IQR)	61 (56–67)	62 (57–67)	62 (57–68)	60 (55–66)	61 (56–67)	60 (55–65)	<0.001
Age (≥65), n (%)	4332 (31)	1747 (34)	670 (38)	992 (28)	475 (32)	448 (24)	<0.001
Male, n (%)	13,418 (97)	4952 (97)	1713 (97)	3480 (97)	1440 (97)	1833 (98)	0.829
Race and ethnicity, n (%)							<0.001
Hispanic	1014 (7)	297 (6)	154 (9)	278 (8)	162 (11)	123 (7)	
Black, nonhispanic	6975 (51)	2627 (52)	822 (47)	1751 (49)	650 (44)	1125 (60)	
White, nonhispanic	5260 (38)	1972 (39)	719 (41)	1390 (39)	626 (42)	553 (29)	
Other/missing	558 (4)	201 (4)	70 (4)	161 (4)	48 (3)	78 (4)	
Region							<0.001
Midwest	2027 (15)	740 (15)	250 (14)	570 (16)	242 (16)	225 (12)	
Northeast	3195 (23)	1304 (26)	371 (21)	741 (21)	335 (23)	444 (24)	
Southeast	4782 (35)	1679 (33)	646 (37)	1363 (38)	482 (32)	612 (33)	
Southwest	1508 (11)	508 (10)	204 (12)	349 (10)	159 (11)	288 (15)	
West	2295 (17)	866 (17)	294 (17)	557 (16)	268 (18)	310 (16)	
Smoking, n (%)							<0.001
Current	6965 (50)	2575 (51)	819 (46)	1719 (48)	768 (52)	1084 (58)	
Past	3912 (28)	1436 (28)	558 (32)	1061 (30)	381 (26)	476 (25)	
Never/Unknown	2930 (21)	1086 (21)	388 (22)	800 (22)	337 (23)	319 (17)	
Alcohol use disorder (recent 12 m), n (%)	2596 (19)	985 (19)	310 (18)	626 (17)	266 (18)	409 (22)	0.001
Drug use and dependence (recent 12 m), n (%)	2232 (16)	847 (17)	228 (13)	490 (14)	249 (17)	418 (22)	<0.001
Homeless (recent 12 m), n (%)	1291 (9)	459 (9)	133 (8)	320 (9)	126 (8)	253 (13)	<0.001
Statin, n (%)	5639 (41)	2232 (44)	825 (47)	1358 (38)	567 (38)	657 (35)	<0.001
Total cholesterol (mg/dl), median (IQR)	172 (147–200)	172 (146–200)	174 (149–201)	175 (151–203)	163 (139–191)	172 (146–198)	<0.001
High-density lipoprotein (mg/dl), median (IQR)	43 (35–54)	44 (35–54)	46 (37–56)	44 (36–54)	41 (33–50)	42 (35–53)	<0.001
Low-density lipoprotein (mg/dl), median (IQR)	97 (76–119)	97 (75–118)	98 (76–121)	99 (80–122)	92 (70–113)	96 (75–117)	<0.001
Triglyceride (mg/dl), median (IQR)	132 (91–199)	134 (92–202)	125 (89–177)	132 (91–196)	136 (92–209)	133 (93–207)	<0.001
Hemoglobin (g/dl), median (IQR)	14 (13–15)	14 (13–15)	14 (13–15)	14 (13–15)	14 (13–15)	14 (13–15)	<0.001
A1C (%), median (IQR)	5.6 (5.2–6.0)	5.6 (5.2–6.0)	5.6 (5.3–6.0)	5.6 (5.2–6.0)	5.7 (5.2–6.2)	5.6 (5.2–6.0)	<0.001
Estimated glomerular filtration rate (ml/min/1.73 m^2^), median (IQR)	72 (60–91)	69 (59–89)	74 (60–92)	74 (60–91)	73 (59–91)	74 (60–93)	<0.001
Obesity (BMI ≥ 30 kg/m^2^), n (%)	3265 (24)	1195 (23)	459 (26)	870 (24)	354 (24)	387 (21)	<0.001
Diabetes (ever), n (%)	3853 (28)	1480 (29)	476 (27)	874 (24)	505 (34)	518 (28)	<0.001
Diabetes (type 2), n (%)	3824 (28)	1470 (29)	468 (27)	871 (24)	504 (34)	511 (27)	<0.001
Hypertension, controlled, with prescriptions, n (%)	5835 (42)	2217 (43)	736 (42)	1432 (40)	648 (44)	802 (43)	0.016
Hypertension, uncontrolled, n (%)	2400 (17)	794 (16)	300 (17)	713 (20)	244 (16)	349 (19)	<0.001
Cardiovascular disease (ever), n (%)	2838 (21)	1116 (22)	362 (21)	580 (16)	387 (26)	393 (21)	<0.001
Hepatitis B virus infection (ever), n (%)	940 (7)	328 (6)	111 (6)	214 (6)	137 (9)	150 (8)	<0.001
Hepatitis C virus infection (ever), n (%)	3184 (23)	1290 (25)	255 (14)	610 (17)	515 (35)	514 (27)	<0.001
Major depression (ever), n (%)	5185 (38)	1920 (38)	692 (39)	1282 (36)	586 (39)	705 (38)	0.062
Comorbidities, n (%)							<0.001
0	2967 (21)	960 (19)	479 (27)	915 (26)	240 (16)	373 (20)	
1	6686 (48)	2420 (47)	847 (48)	1815 (51)	677 (46)	927 (49)	
≥2	4154 (30)	1717 (34)	439 (25)	850 (24)	569 (38)	579 (31)	
CD4 (cells/uL), median (IQR)	541 (348–760)	554 (363–776)	610 (409–818)	559 (370–773)	498 (299–716)	434 (253–661)	<0.001
Viral load (copies/mL), median (IQR)	20 (20–40)	20 (20–40)	20 (20–40)	20 (20–41)	20 (20–40)	40 (20–385)	<0.001
Virologically suppressed, n (%)^b^	9613 (76)	3760 (79)	1306 (82)	2493 (76)	1026 (79)	1028 (62)	<0.001
Low-level viremia, n (%)^b^	926 (7)	345 (7)	95 (6)	225 (7)	88 (7)	173 (10)	<0.001
VACS index 2.0, median (IQR)	50 (40–63)	49 (39–63)	56 (48–66)	45 (36–57)	51 (41–65)	53 (42–68)	<0.001
Number of ARV regimens used before, median (IQR)	5 (2–10)	5 (3–10)	4 (2–9)	5 (2–9)	7 (3–12)	7 (3–13)	<0.001
Time on ARVs (years), median (IQR)	12 (7–18)	12 (7–18)	13 (8–19)	11 (6–17)	12 (6–18)	12 (6–18)	<0.001
Duration of preceding regimen (months), median (IQR)	60 (21–106)	65 (29–110)	57 (3–121)	59 (17–106)	63 (27–102)	50 (17–88)	<0.001
Non-ARV co-medications (recent 12 m), median (IQR)	9 (5–15)	9 (5–15)	8 (4–14)	8 (4–13)	10 (6–16)	9 (5–14)	<0.001

*DTG* dolutegravir, *BIC* bictegravir, *EVG* elvitegravir, *RAL* raltegravir, *DRV* darunavir, *PWH* persons with HIV, *CD4* clusters of differentiation 4, *VACS* the Veterans Aging Cohort Study, *ARV* antiretroviral, *ART* antiretroviral therapy, *IQR* interquartile range, *Rx* prescription, *BMI* body mass index

^a^The p-value, based on a Chi-squared test for categorical variables and an analysis of variance (ANOVA) test for numerical variables, assesses the difference of each variable across regimen groups

^b^Percentages are calculated based on individuals with non-missing VL at baseline

Regardless of regimen or ART experience, most individuals were male (95–97% across all treatment groups). The median age at baseline was 59 years (22% ≥ 65 years) for ART-naïve individuals and 61 years (31% ≥ 65 years) for ART-experienced. Greater proportions of ART-experienced individuals had two or more comorbidities (30 vs. 21%), and a higher prevalence of diabetes (28 vs. 21%), cardiovascular disease (21 vs. 11%), and hepatitis C infection (23 vs. 13%) compared to ART-naïve individuals. Overall, baseline CD4 count was lower (median 318 vs. 541 cells/uL) for ART-naïve individuals than ART-experienced individuals, while baseline VL was higher (median 46,850 copies/mL vs. 20 copies/mL) for ART-naïve individuals compared to ART-experienced individuals. The baseline VACS index 2.0 medians were 66 and 50 for the ART-naïve and ART-experienced. The median number of non-ARV co-medications individuals were exposed to within a year prior to index date was lower (7 vs. 9) in the ART-naïve group compared to ART-experienced individuals.

Among ART-naïve individuals, 27% PWH on BIC and 24% on DRV were older than 65 years compared to DTG (22%). Current smoker was 42% for BIC and 62% for RAL compared to 53% for DTG. Those on RAL and DRV had more comorbidities, lower CD4, higher viral load and higher VAC index 2.0 compared to those initiating ART with DTG (58 and 49% vs 45% for ≥1 comorbidities; 294 and 159 vs 334 cells/uL for median CD4 count; 38,810 and 70,800 vs 46,800 copies/mL for median VL; 70 and 71 vs 66 for median VACS index 2.0).

In the ART-experienced group, more PWH on BIC (38%) and DTG (34%) were older than 65 years compared to those on DRV (24%). A higher proportion on DRV were current smoker (58 vs 51% for DTG) and had alcohol use disorder (22 vs 19% for DTG), drug use or dependence (22 vs 17% for DTG), and homelessness (13 vs 9% for DTG). Compared to those on DTG, individuals on RAL and DRV had a higher rate of hepatitis B virus infection (9 and 8% vs 6%), hepatitis C virus infection (35 and 27% vs 25%), lower median CD4 count (498 and 434 vs 554 cells/uL), lower proportions being suppressed (69 and 55% vs 74%), higher VACS index 2.0 (51 and 53 vs 49), and more non-ARV co-medications (10 and 9 vs 9). ART-experienced individuals have been on antiretrovirals (ARVs) for about 11–13 years, 55–74% being virologically suppressed and 5–9% having low-level viremia across all drug regimens.

### Effectiveness outcomes

**Fig. 2:**
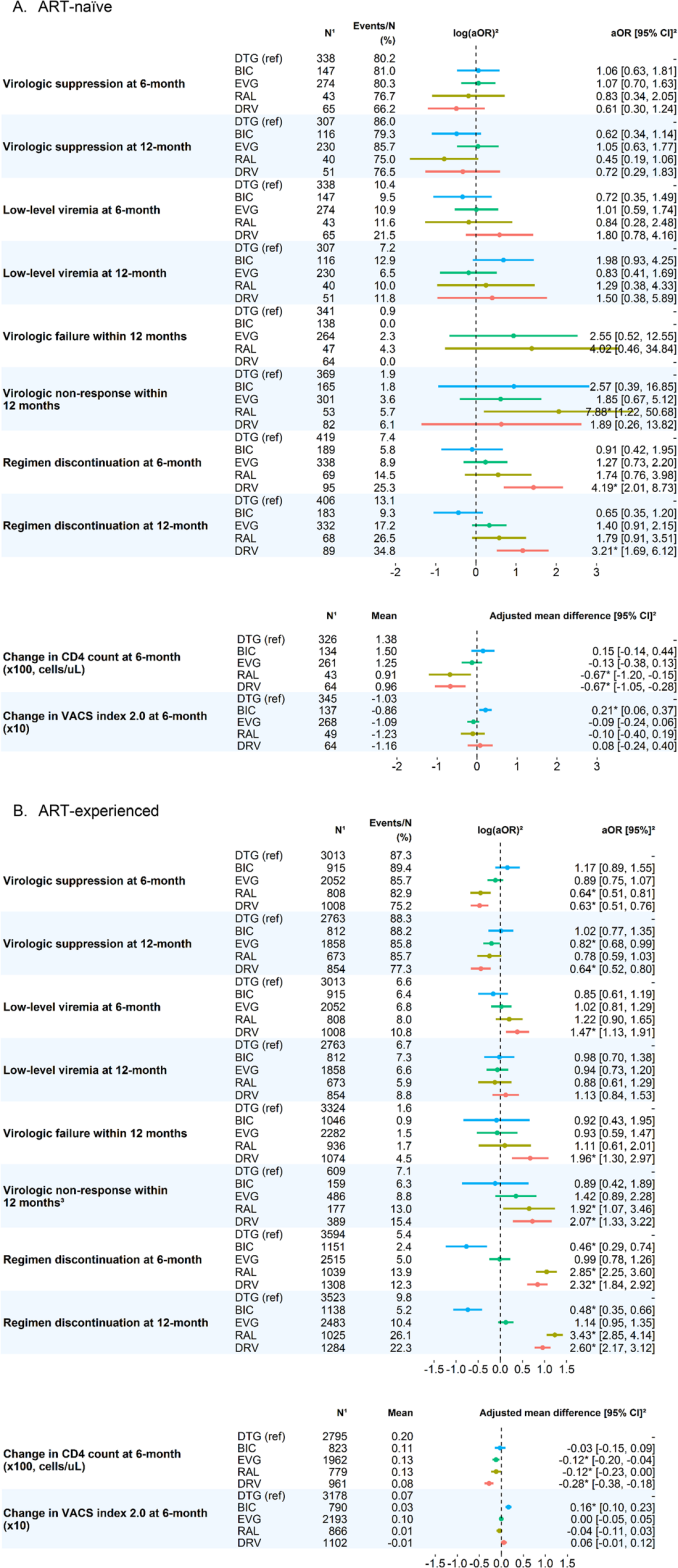
Comparison of treatment outcomes for those receiving BIC-, EVG-, RAL-, and DRV-based 3-drug regimens compared to those receiving DTG-based 3-drug regimen among A. ART-naïve and B. ART-experienced PWH. ^1^N represents the number of persons in each treatment group with complete information on variables used in the outcome model. ^2^Estimates and confidence intervals were calculated from inverse-probability weighted models, adjusted for age, sex, race and/or ethnicity, region, smoking, alcohol use disorder, drug use and dependence, homelessness, baseline low-density lipoprotein, baseline CD4 count, baseline VL, baseline VACS 2.0 index, and years on ART regimen for ART-experienced. ^3^Virologic non-response for ART-experienced was defined for individuals who were suppressed at baseline. *DTG* dolutegravir, *BIC* bictegravir, *EVG* elvitegravir, *RAL* raltegravir, *DRV* darunavir, *PWH* people with HIV, *ART* antiretroviral therapy, *aOR* adjusted odds ratio, *CI* confidence interval, *VACS* the Veterans Aging Cohort Study, *CD4* clusters of differentiation 4, *VL* viral load

Among ART-naïve individuals (Fig. 2, Panel A), there were no statistical differences in virologic suppression and low-level viremia across regimen groups at 6 and 12 months from regimen initiation. Within 12 months, there were no statistical differences in odds of experiencing virologic failure between DTG, EVG, and RAL (aORs for BIC and DRV not reported due to few events). Compared to DTG, only individuals on RAL had statistically higher odds of experiencing non-response in the first 12 months of treatment (aOR 7.88, 95% CI 1.22–50.68). Those on DRV were more likely to discontinue regimen by 6 months (aOR 4.19, 95% CI 2.01–8.73) and 12 months (aOR 3.21, 95% CI 1.69–6.12) compared to DTG. Mean CD4 count increased in the first 6 months of treatment with ART among all regimen groups, but those on RAL and DRV had a smaller increase compared to those on DTG (adjusted mean difference [95% CI], −67 cells/uL [−120 to −15] for RAL vs DTG, −67 cells/uL [−105 to −28] for DRV vs DTG). Mean VACS index 2.0 decreased within the first 6 months of treatment, regardless of regimens, with those on BIC experiencing smaller adjusted mean decreases compared to those on DTG (adjusted mean difference [95% CI], 2.1 [0.6–3.7]).

Among ART-experienced individuals (Fig. 2, Panel B), compared to those on DTG, those on RAL and DRV were less likely to be suppressed at 6 months (RAL vs DTG: aOR 0.64, 95% CI 0.51–0.81; DRV vs DTG: aOR 0.63, 95% CI 0.51–0.76) and those on EVG and DRV were less likely suppressed at 12 months (EVG vs DTG: aOR 0.82, 95% CI 0.68–0.99; DRV vs DTG: aOR 0.64, 95% CI 0.52–0.80). Those on DRV were more likely to have low-level viremia at 6 months (DRV vs DTG: aOR 1.47, 95% CI 1.13–1.91). Those on DRV were more likely to have virologic failure within 12 months (aOR 1.96, 95% CI 1.30–2.97). Those on RAL and DRV were more likely to experience non-response within 12 months (RAL vs DTG: aOR 1.92, 95% CI 1.07–3.46; DRV vs DTG: aOR 2.07, 95% CI 1.33–3.22) among unsuppressed individuals. Compared to DTG, the mean increase in CD4 count was 12 [95% CI 4–20], 12 [95% CI 0–23], and 28 [95% CI 18–38] cells/uL lower among those on EVG, RAL, and DRV at 6 months. VACS index 2.0 improved to a greater extent among those on BIC compared to those on DTG (adjusted mean difference [95% CI], 1.6 [1.0–2.3]).

Compared to individuals taking DTG-based regimens, those on RAL and DRV were more likely to discontinue ART within 6 months (RAL vs DTG: aOR 2.85, 95% CI 2.25–3.60; DRV vs DTG: aOR 2.32, 95% CI 1.84–2.92) and within 12 months (RAL vs DTG: aOR 3.43, 95% CI 2.85–4.14; DRV vs DTG: aOR 2.60, 95% CI 2.17–3.12). Discontinuation by 6 and 12 months was lower for those on BIC compared to DTG (6-month aOR 0.46, 95% CI 0.29–0.74; 12-month aOR 0.48, 95% CI 0.35–0.66). Reason for discontinuation was not directly reported in the EHR data. Discontinuations of baseline regimens potentially attributed to regimen simplification were identified (Supplemental Table 6), as switches from multiple-tablet baseline regimens to single-tablet regimens, as well as reductions from a 3-drug regimen to a 2-drug regimen, and switching from a boosted regimen (4 drugs total) to a regimen with 3 or fewer drugs. Among those discontinuing baseline regimens, 22% of ART-naïve and 27% ART-experienced on DTG, 39% of ART-naïve and 40% of ART-experienced on RAL, and 77% of ART-naïve and 45% of ART-experienced on DRV switched from a multi-tablet regimen to a single-tablet regimen when they discontinued their baseline regimen. Discontinuing a boosted regimen (EVG- and DRV-based regimens) to move to a 2- or 3-drug non-boosted regimen was also common (75% of ART-naïve and ART-experienced discontinuing a cobicistat boosted EVG regimen and 77% of ART-naïve and 45% of ART-experienced discontinuing a boosted DRV-based regimen). Simplification from a 3-drug (boosted or unboosted) regimen to a 2-drug regimen occurred rarely in the ART-naïve group (n = 3 switches) but more frequently in the ART-experienced group (8% of DTG discontinuations, 11% BIC, 1% EVG, 3% RAL, and 2% DRV).

Outcomes of subgroup analyses based on age group (50–64 years and ≥65 years) and hepatitis C virus (HCV and no HCV) among ART-experienced individuals (Supplemental Figs. 2 and 3), were comparable to findings from the primary analysis.

Sensitivity analyses were performed to assess the robustness of our findings and yielded results that were consistent with those obtained from the primary analysis (Supplement Figs. 4–6).

## Discussion

In a large national sample of older veterans with HIV who were on various ART regimens, individuals on DTG- and BIC-based regimens experienced similar virologic and immunologic responses to treatment among both ART-naïve and ART-experienced individuals across endpoints. Responses to treatment tended to be lower among those taking RAL- and DRV-based regimens, even after accounting for baseline differences in characteristics. Specifically, among ART-naïve PWH initiating treatment, compared to those on DTG-based regimens, immune response within the first 6 months was lower for those taking RAL and DRV. Among ART-experienced PWH, those treated with RAL-and DRV-based regimens were less likely to achieve or maintain suppression and experienced lower gains in CD4 within the first year of treatment; those on DRV-based regimens were also more likely to experience virologic non-response and failure. Treatment experienced individuals on boosted EVG-based regimens also had lower suppression and smaller changes in CD4 compared to those on DTG-based regimens.

Our findings on virologic outcomes were consistent with the results of previous randomized clinical trials. In clinical trials, bictegravir/emtricitabine/tenofovir alafenamide (BIC/FTC/TAF) demonstrated non-inferiority to dolutegravir/abacavir/lamivudine in terms of virologic suppression at week 48 in both treatment-naïve and treatment-experienced populations [22, 23]. In the FLAMINGO trial, participants receiving DTG (with tenofovir-emtricitabine or abacavir-lamivudine) were more likely to achieve virologic suppression at week 48 than those receiving DRV plus ritonavir among ART-naïve adults [24].

Several observational studies and meta-analyses have demonstrated that INSTI-based regimens, particularly second generation INSTIs of DTG and BIC, were associated with longer treatment duration and better virologic suppression compared to non-INSTI regimens [25–30]. Fewer studies have compared the effectiveness of 3-drug ART regimens between INSTIs. D’Arminio Monforte et al. [31] showed that DTG had a lower risk of treatment failure than RAL and DRV at 12 months among ART-naïve patients, with no significant difference in the risk of discontinuation. Mills et al. [32] found that ART-naïve PWH on DTG had better virologic outcomes than RAL and DRV but had an increased virologic failure risk comparable to EVG. Brehm et al. [33] found no significant differences in virologic suppression between DTG, EVG, and RAL at 12 months, both in ART-naïve and ART-experienced patients. Consistent with these studies, our findings suggest that DTG-based regimens had a higher likelihood of virologic suppression or lower virologic failure than EVG, RAL, and DRV among ART-experienced patients.

Data on virologic and immunologic treatment responses specifically among older PWH are limited. A pooled analysis using data from 6 phase III/IIIb clinical trials assessing the efficacy and safety of dolutegravir-based ART found that in both treatment-naive and treatment-experienced study participants, response rates to dolutegravir-based ART were similar when compared between age groups: <50, ≥50 to <65, and ≥65 years [34]. Response rates to non-dolutegravir-based ART were numerically lower in the ≥65 years group; however, participant numbers were too low to draw any meaningful conclusions. Efficacy between DTG- and non-DTG regimens among the older age groups were not directly compared. A phase 3b single‐arm trial evaluating virologically suppressed PWH aged ≥65 years switching from EVG/c/emtricitabine/tenofovir alafenamide or a tenofovir disoproxil fumarate‐based regimen to BIC/emtricitabine/tenofovir alafenamide found high rates of suppression and stable CD4 counts through 96 weeks of follow up [35]. Comparative efficacy among older PWH in clinical trials and real-world effectiveness of 3-drug regimens restricted to older PWH are currently data gaps in the literature.

PWH taking DRV- and RAL-based regimens were more likely to discontinue their regimens by 6- or 12-months compared to DTG-based regimens among both the ART-naïve and ART-experienced populations. Worse virologic or immunologic response to treatment necessitating changes to the baseline regimen may partially account for the higher discontinuation rates compared to those on DTG-based regimens. In contrast, despite comparable virologic and immunologic responses to treatment, individuals on BIC-based regimens were less likely to discontinue within 6- or 12-months of treatment among ART-experienced PWH.

It is a limitation of the data that reasons for regimen changes and discontinuations are not consistently documented in the EHR data. Looking at the regimens proceeding discontinuations, simplification may have been a common driver for treatment change, with a large proportion of those on DTG-, RAL- and DRV-based regimens switching from multi-tablet to single-tablet regimens. BIC- and EVG-based regimens are only available in fixed dose combination single-tablet regimens. In addition to the number of pills, simplification by reducing the total number of drugs in the regimen, particularly the elimination of boosting agents, was another common scenario among discontinuations. Complete 2-drug regimens for HIV treatment (approved regimens: DTG/lamivudine and DTG/rilpivirine) were newly approved during the time period of the study, but simplification to a 2-drug was another form of simplification observed, particularly in the ART-experienced population and among those already on 2nd generation INSTIs (DTG and BIC). In an aging population with higher likelihood of multiple comorbidities and associated polypharmacy, reducing drug and pill burdens are important considerations to limit the risks of adherence issues, drug-drug interactions, and drug toxicities [36].

This study has additional limitations. Given differences in veteran demographic and clinical characteristics compared with the general US population, as well as differences in care delivery between VHA and non-VHA systems, findings from this study may not be generalizable to other groups. Additionally, we were not able to evaluate adherence or resistance based on the available data, both of which may impact response to treatment and discontinuations. Further, while we employed methods to control for potential confounders that may affect the results, missing data and unknown confounders that were not included in the EHR could result in residual confounding. Of note, those taking RAL- and DRV-based regimens were more likely to have a complex medical presentation, including more frequent comorbid conditions and associated polypharmacy. While the statistical methods used attempted to account for potential channeling bias, residual confounding could still bias observed results. COVID-19 may have impacted clinical practices towards the end of the study period, with visits not being scheduled as regularly as during normal circumstances. Last, with a follow-up of up to 1 year, we were not able to compare the regimens in the long term.

## Conclusions

Among both ART-naïve and ART-experienced veterans of age ≥50 in the VACS cohort, those starting a DTG-based 3-drug regimen had comparable or favorable treatment responses to those starting BIC-, EVG-, RAL-, and DRV-based 3-drug regimens.

## Supplementary Information

Additional file 1

## Data Availability

No datasets were generated or analysed during the current study.

## Declarations

### Disclaimer

The contents do not represent the views of the U.S. Department of Veterans Affairs or the United States Government.

## References

[1] Dow DE, Bartlett JA. Dolutegravir, the second-generation of integrase strand transfer inhibitors (INSTIs) for the treatment of HIV. Infect Dis Ther. 2014;3(2):83–102. 10.1007/s40121-014-0029-7. 25134686 10.1007/s40121-014-0029-7 PMC4269626

[2] Mbhele N, Chimukangara B, Gordon M. HIV-1 integrase strand transfer inhibitors: a review of current drugs, recent advances and drug resistance. Int J Antimicrob Agents. 2021;57(5):106343. 10.1016/j.ijantimicag.2021.106343. 33852932 10.1016/j.ijantimicag.2021.106343

[3] Zhabokritsky A, Szadkowski L, Burchell AN, et al. Immunological and virological response to initial antiretroviral therapy among older people living with HIV in the Canadian Observational Cohort (CANOC). HIV Med. 2021;22(8):759–69. 10.1111/hiv.13125. 34075683 10.1111/hiv.13125

[4] Richterman A, Sax PE. Antiretroviral therapy in older people with HIV. Curr Opin HIV AIDS. 2020;15(2):118–25. 10.1097/COH.0000000000000614. 31990705 10.1097/COH.0000000000000614

[5] Dunn-Walters D, Martin V, Abdulla A. The immune system. In: Rai G, Abdulla A, editors. The biology of ageing. CRC Press; 2013. p. 35–49: chap 4.

[6] Montecino-Rodriguez E, Berent-Maoz B, Dorshkind K. Causes, consequences, and reversal of immune system aging. J Clin Invest. 2013;123(3):958–65. 10.1172/JCI64096. 23454758 10.1172/JCI64096 PMC3582124

[7] Cai CW, Sereti I. Residual immune dysfunction under antiretroviral therapy. Semin Immunol. 2021;51:101471. 10.1016/j.smim.2021.101471. 33674177 10.1016/j.smim.2021.101471 PMC8410879

[8] Vider E, Gavioli EM. Clinical safety considerations of integrase strand transfer inhibitors in the older population living with HIV. Drugs Aging. 2021;38(11):967–75. 10.1007/s40266-021-00894-y. 34494229 10.1007/s40266-021-00894-y

[9] Chawla A, Wang C, Patton C, et al. A review of long-term toxicity of antiretroviral treatment regimens and implications for an aging population. Infect Dis Ther. 2018;7(2):183–95. 10.1007/s40121-018-0201-6. 29761330 10.1007/s40121-018-0201-6 PMC5986685

[10] Smit M, Brinkman K, Geerlings S, et al. Future challenges for clinical care of an ageing population infected with HIV: a modelling study. Lancet Infect Dis. 2015;15(7):810–8. 10.1016/S1473-3099(15)00056-0. 26070969 10.1016/S1473-3099(15)00056-0 PMC4528076

[11] Jourjy J, Dahl K, Huesgen E. Antiretroviral treatment efficacy and safety in older HIV-infected adults. Pharmacotherapy. 2015;35(12):1140–51. 10.1002/phar.1670. 26684554 10.1002/phar.1670

[12] Fultz SL, Skanderson M, Mole LA, et al. Development and verification of a “virtual” cohort using the National VA Health Information System. Med Care. 2006;44(8 Suppl 2):S25-30. 10.1097/01.mlr.0000223670.00890.74. 16849965 10.1097/01.mlr.0000223670.00890.74

[13] Justice AC, Dombrowski E, Conigliaro J, et al. Veterans Aging Cohort Study (VACS): overview and description. Med Care. 2006;44(8 Suppl 2):S13-24. 10.1097/01.mlr.0000223741.02074.66. 16849964 10.1097/01.mlr.0000223741.02074.66 PMC3049942

[14] Tate JP, Sterne JAC, Justice AC, Veterans Aging Cohort S, the Antiretroviral Therapy Cohort C. Albumin, white blood cell count, and body mass index improve discrimination of mortality in HIV-positive individuals. AIDS. 2019;33(5):903–12. 10.1097/QAD.0000000000002140. 10.1097/QAD.0000000000002140 PMC6749990 30649058

[15] Austin PC. An introduction to propensity score methods for reducing the effects of confounding in observational studies. Multivar Behav Res. 2011;46(3):399–424. 10.1080/00273171.2011.568786. 10.1080/00273171.2011.568786 PMC3144483 21818162

[16] Austin PC, Stuart EA. Moving towards best practice when using inverse probability of treatment weighting (IPTW) using the propensity score to estimate causal treatment effects in observational studies. Stat Med. 2015;34(28):3661–79. 10.1002/sim.6607. 26238958 10.1002/sim.6607 PMC4626409

[17] Cole SR, Hernan MA. Constructing inverse probability weights for marginal structural models. Am J Epidemiol. 2008;168(6):656–64. 10.1093/aje/kwn164. 18682488 10.1093/aje/kwn164 PMC2732954

[18] Lee BK, Lessler J, Stuart EA. Weight trimming and propensity score weighting. PLoS ONE. 2011;6(3):e18174. 10.1371/journal.pone.0018174. 21483818 10.1371/journal.pone.0018174 PMC3069059

[19] Austin PC. Balance diagnostics for comparing the distribution of baseline covariates between treatment groups in propensity-score matched samples. Stat Med. 2009;28(25):3083–107. 10.1002/sim.3697. 19757444 10.1002/sim.3697 PMC3472075

[20] Seaman SR, White IR. Review of inverse probability weighting for dealing with missing data. Stat Methods Med Res. 2011;22(3):278–95. 10.1177/0962280210395740. 21220355 10.1177/0962280210395740

[21] Robins JM, Hernan MA, Brumback B. Marginal structural models and causal inference in epidemiology. Epidemiology. 2000;11(5):550–60. 10.1097/00001648-200009000-00011. 10955408 10.1097/00001648-200009000-00011

[22] Molina JM, Ward D, Brar I, et al. Switching to fixed-dose bictegravir, emtricitabine, and tenofovir alafenamide from dolutegravir plus abacavir and lamivudine in virologically suppressed adults with HIV-1: 48 week results of a randomised, double-blind, multicentre, active-controlled, phase 3, non-inferiority trial. Lancet HIV. 2018;5(7):e357–65. 10.1016/S2352-3018(18)30092-4. 29925489 10.1016/S2352-3018(18)30092-4

[23] Gallant J, Lazzarin A, Mills A, et al. Bictegravir, emtricitabine, and tenofovir alafenamide versus dolutegravir, abacavir, and lamivudine for initial treatment of HIV-1 infection (GS-US-380-1489): a double-blind, multicentre, phase 3, randomised controlled non-inferiority trial. Lancet. 2017;390(10107):2063–72. 10.1016/S0140-6736(17)32299-7. 28867497 10.1016/S0140-6736(17)32299-7

[24] Clotet B, Feinberg J, van Lunzen J, et al. Once-daily dolutegravir versus darunavir plus ritonavir in antiretroviral-naive adults with HIV-1 infection (FLAMINGO): 48 week results from the randomised open-label phase 3b study. Lancet. 2014;383(9936):2222–31. 10.1016/S0140-6736(14)60084-2. 24698485 10.1016/S0140-6736(14)60084-2

[25] Mayer S, Rayeed N, Novak RM, et al. INSTI-based initial antiretroviral therapy in adults with HIV, the HIV Outpatient Study, 2007–2018. AIDS Res Hum Retroviruses. 2021;37(10):768–75. 10.1089/AID.2020.0286. 34030459 10.1089/AID.2020.0286 PMC12380107

[26] Jacobson K, Ogbuagu O. Integrase inhibitor-based regimens result in more rapid virologic suppression rates among treatment-naive human immunodeficiency virus-infected patients compared to non-nucleoside and protease inhibitor-based regimens in a real-world clinical setting: a retrospective cohort study. Medicine. 2018;97(43):e13016. 10.1097/MD.0000000000013016. 30412140 10.1097/MD.0000000000013016 PMC6221636

[27] Lu H, Cole SR, Westreich D, et al. Clinical effectiveness of integrase strand transfer inhibitor-based antiretroviral regimens among adults with human immunodeficiency virus: a collaboration of cohort studies in the United States and Canada. Clin Infect Dis. 2021;73(7):e1408–14. 10.1093/cid/ciaa1037. 32780095 10.1093/cid/ciaa1037 PMC8492356

[28] Lu H, Cole SR, Westreich D, et al. Virologic outcomes among adults with HIV using integrase inhibitor-based antiretroviral therapy. AIDS. 2022;36(2):277–86. 10.1097/QAD.0000000000003069. 34934020 10.1097/QAD.0000000000003069 PMC9048218

[29] Snedecor SJ, Radford M, Kratochvil D, Grove R, Punekar YS. Comparative efficacy and safety of dolutegravir relative to common core agents in treatment-naive patients infected with HIV-1: a systematic review and network meta-analysis. BMC Infect Dis. 2019;19(1):484. 10.1186/s12879-019-3975-6. 31146698 10.1186/s12879-019-3975-6 PMC6543679

[30] Nickel K, Halfpenny NJA, Snedecor SJ, Punekar YS. Comparative efficacy, safety and durability of dolutegravir relative to common core agents in treatment-naive patients infected with HIV-1: an update on a systematic review and network meta-analysis. BMC Infect Dis. 2021;21(1):222. 10.1186/s12879-021-05850-0. 33637050 10.1186/s12879-021-05850-0 PMC7908737

[31] d’Arminio Monforte A, Cozzi-Lepri A, Di Biagio A, et al. Durability of first-line regimens including integrase strand transfer inhibitors (INSTIs): data from a real-life setting. J Antimicrob Chemother. 2019;74(5):1363–7. 10.1093/jac/dky566. 30698801 10.1093/jac/dky566

[32] Mills AM, Brunet L, Fusco JS, et al. Virologic outcomes among ART-naive individuals initiating Dolutegravir, Elvitegravir, Raltegravir or Darunavir: an Observational Study. Infect Dis Ther. 2020;9(1):41–52. 10.1007/s40121-019-00274-5. 31701370 10.1007/s40121-019-00274-5 PMC7054577

[33] Brehm TT, Franz M, Hufner A, et al. Safety and efficacy of elvitegravir, dolutegravir, and raltegravir in a real-world cohort of treatment-naive and -experienced patients. Medicine. 2019;98(32):e16721. 10.1097/MD.0000000000016721. 31393378 10.1097/MD.0000000000016721 PMC6708907

[34] Spinelli F, Prakash M, Slater J, et al. Dolutegravir-based regimens in treatment-naive and treatment-experienced aging populations: analyses of 6 phase III clinical trials. HIV Res Clin Pract. 2021;22(2):46–54. 10.1080/25787489.2021.1941672. 34180785 10.1080/25787489.2021.1941672

[35] Maggiolo F, Rizzardini G, Molina JM, et al. Bictegravir/emtricitabine/tenofovir alafenamide in older individuals with HIV: results of a 96-week, phase 3b, open-label, switch trial in virologically suppressed people ≥65 years of age. HIV Med. 2023;24(1):27–36. 10.1111/hiv.13319. 35527425 10.1111/hiv.13319 PMC10083930

[36] Back D, Marzolini C. The challenge of HIV treatment in an era of polypharmacy. J Int AIDS Soc. 2020;23(2):e25449. 10.1002/jia2.25449. 32011104 10.1002/jia2.25449 PMC6996317

