# Supplementary material for: Effectiveness of dolutegravir-based regimens compared to raltegravir-, elvitegravir-, bictegravir, and darunavir-based regimens among older adults with HIV in the Veterans Aging Cohort Study (VACS)
Source: AIDS Res Ther. 2024 Dec 21;21:96. doi: 10.1186/s12981-024-00681-w (PMC11662819; doi:10.1186/s12981-024-00681-w)
Supplement: Supplementary file 1 — Additional file 1 [file 12981_2024_681_MOESM1_ESM.docx]

**Supplemental Material for**

**Effectiveness of dolutegravir-based regimens compared to raltegravir-, elvitegravir-, bictegravir-, and darunavir-based regimens among older adults with HIV in the Veterans Aging Cohort Study (VACS)**

**Table of Contents**

[Supplemental Table 1. ART regimens included in each treatment group 2](#_Toc182913587)

[Supplemental Table 2. The nucleoside reverse transcriptase inhibitors used with the core agents 4](#_Toc182913588)

[Supplemental Table 3. Missing values in baseline and outcome variables 5](#_Toc182913589)

[Supplemental Table 4. Deaths within 6 and 12 months 6](#_Toc182913590)

[Supplemental Table 5. Initiation year of ART regimens 7](#_Toc182913591)

[Supplemental Table 6. Exploration of reasons for discontinuing regimens 8](#_Toc182913592)

[Supplemental Figure 1. Measuring windows 10](#_Toc182913593)

[Supplemental Figure 2. Effectiveness for those receiving BIC-, EVG-, RAL-, and DRV-based 3-drug regimens compared to those receiving DTG-based 3-drug regimen among ART-experienced PWH stratified by age group 11](#_Toc182913594)

[Supplemental Figure 3. Effectiveness for those receiving BIC-, EVG-, RAL-, and DRV-based 3-drug regimens compared to those receiving DTG-based 3-drug regimen among ART-experienced PLWH stratified by hepatitis C virus (HCV) 13](#_Toc182913595)

[Supplemental Figure 4. Comparison of treatment outcomes for those receiving BIC-, EVG-, RAL-, and DRV-based 3-drug regimens compared to those receiving DTG-based 3-drug regimen among A. ART-naïve and B. ART-experienced PWH where missing values were treated as a separate category in the weighting model 15](#_Toc182913596)

[Supplemental Figure 5. Comparison of methods 17](#_Toc182913597)

[Supplemental Figure 6. Comparison of treatment outcomes for those receiving BIC-based 3-drug regimens compared to those receiving DTG-based 3-drug regimen among A. ART-naïve and B. ART-experienced PWH during the period after the approval of BIC 19](#_Toc182913598)

# Supplemental Table 1. ART regimens included in each treatment group

Counts and percentages of ART regimens

|  |  | **n (%)** | **Single-tablet, n** |
| --- | --- | --- | --- |
| **DTG (N=5800)** | 3TC_ABC_DTG | 3623 (62) | Triumeq: 3035 |
|  | DTG_FTC_TAF | 955 (16) | DTG+Descovy: 954 |
|  | DTG_FTC_TDF | 884 (15) | DTG+Truvada: 870 |
|  | 3TC_ABC_DTG_RTV | 135 (2) |  |
|  | DTG_FTC_RTV_TDF | 69 (1) |  |
|  | 3TC_AZT_DTG | 40 (1) |  |
|  | DTG_FTC_RTV_TAF | 40 (1) |  |
|  | 3TC_DTG_TDF | 23 (0) |  |
|  | Other DTG-based 3DR | 31 (1) |  |
|  |  |  |  |
| **BIC (N=2081)** | BIC_FTC_TAF | 2028 (97) | Biktarvy: 2081 |
|  | BIC_FTC_RTV_TAF | 53 (3) |  |
|  |  |  |  |
| **EVG (N=4159)** | COBIC_EVT_FTC_TAF | 2735 (66) | Genvoya: 2735 |
|  | COBIC_EVT_FTC_TDF | 1424 (34) | Stribild: 1423 |
|  |  |  |  |
| **RAL (N=1607)** | FTC_RAL_TDF | 803 (50) | RAL+Truvada: 791 |
|  | 3TC_ABC_RAL | 278 (17) | RAL+Epzicom: 213 |
|  | FTC_RAL_TAF | 263 (16) | RAL+ Descovy: 262 |
|  | FTC_RAL_RTV_TDF | 80 (5) |  |
|  | 3TC_AZT_RAL | 79 (5) |  |
|  | 3TC_RAL_TDF | 30 (2) |  |
|  | 3TC_ABC_RAL_RTV | 21 (1) |  |
|  | ABC_RAL_TDF | 13 (1) |  |
|  | Other RAL-based 3DR | 40 (3) |  |
|  |  |  |  |
| **DRV (N=2055)** | DRV_FTC_RTV_TDF | 872 (42) | DRV+RTV+Truvada: 859 |
|  | 3TC_ABC_DRV_RTV | 326 (16) | DRV+RTV+Epzicom: 266 |
|  | COBIC_DRV_FTC_TAF | 217 (11) | Symtuza: 22 |
|  | DRV_FTC_RTV_TAF | 193 (9) | DRV+RTV+ Descovy: 191 |
|  | DRV_FTC_TDF | 116 (6) | DRV+Truvada: 115 |
|  | COBIC_DRV_FTC_TDF | 109 (5) | Prezcobix + Truvada: 107 |
|  | 3TC_AZT_DRV_RTV | 59 (3) |  |
|  | 3TC_ABC_COBIC_DRV | 48 (2) |  |
|  | 3TC_ABC_DRV | 22 (1) |  |
|  | 3TC_DRV_RTV_TDF | 19 (1) |  |
|  | ABC_DRV_RTV_TDF | 12 (1) |  |
|  | Other DRV-based 3DR | 62 (3) |  |

ART drug names and abbreviations

| Abbreviation | Generic Name | Drug Class |
| --- | --- | --- |
| COBIC | COBICISTAT | Booster |
| RTV | RITONAVIR | Booster |
| ENF | ENFUVIRTIDE | Entry |
| FTR | FOSTEMSAVIR | Entry |
| MAR | MARAVIROC | Entry |
| DTG | DOLUTEGRAVIR | INSTI |
| EVT | ELVITEGRAVIR | INSTI |
| RAL | RALTEGRAVIR | INSTI |
| DLV | DELAVIRDINE | NNRTI |
| DOR | DORAVIRINE | NNRTI |
| EFV | EFAVIRENZ | NNRTI |
| ETA | ETRAVIRINE | NNRTI |
| NVP | NEVIRAPINE | NNRTI |
| RPV | RILPIVIRINE | NNRTI |
| ABC | ABACAVIR | NRTI |
| ADE | ADEFOVIR | NRTI |
| DDI | DIDANOSINE | NRTI |
| FTC | EMTRICITABINE | NRTI |
| 3TC | LAMIVUDINE | NRTI |
| D4T | STAVUDINE | NRTI |
| TDF | TENOFOVIR DISOPROXIL FUMARATE | NRTI |
| TAF | TENOFOVIR ALAFENAMIDE FUMARATE | NRTI |
| DDC | ZALCITABINE | NRTI |
| AZT | ZIDOVUDINE | NRTI |
| AMP | AMPRENAVIR | PI |
| ATV | ATAZANAVIR | PI |
| EVO | ATAZANAVIR/COBICISTAT | PI |
| PREZ | COBICISTAT/DARUNAVIR | PI |
| DAR | DARUNAVIR | PI |
| FOS | FOSAMPRENAVIR | PI |
| IDV | INDINAVIR | PI |
| KAL | LOPINAVIR/RITONAVIR | PI |
| NFV | NELFINAVIR | PI |
| INV | SAQUINAVIR | PI |
| TPV | TIPRANAVIR | PI |

INSTI: integrase strand transfer inhibitor, NNRTI: non-nucleoside reverse transcriptase inhibitor, NRTI: nucleoside reverse transcriptase inhibitor, PI: protease inhibitor

# Supplemental Table 2. The nucleoside reverse transcriptase inhibitors used with the core agents

| **n (%)** | **N** | **ABC** | **ADE** | **DDI** | **FTC** | **3TC** | **D4T** | **TDF** | **TAF** | **AZT** |
| --- | --- | --- | --- | --- | --- | --- | --- | --- | --- | --- |
| **ART-naive** | | | | | | | | | | |
| DTG | 703 | 437 (62) | 0 (0) | 0 (0) | 261 (37) | 441 (63) | 0 (0) | 113 (16) | 152 (22) | 2 (0) |
| BIC | 316 | 0 (0) | 0 (0) | 0 (0) | 316 (100) | 0 (0) | 0 (0) | 0 (0) | 316 (100) | 0 (0) |
| EVG | 579 | 0 (0) | 0 (0) | 0 (0) | 579 (100) | 0 (0) | 0 (0) | 272 (47) | 307 (53) | 0 (0) |
| RAL | 121 | 11 (9) | 0 (0) | 0 (0) | 106 (88) | 14 (12) | 0 (0) | 97 (80) | 10 (8) | 4 (3) |
| DRV | 176 | 18 (10) | 0 (0) | 0 (0) | 151 (86) | 25 (14) | 0 (0) | 124 (70) | 29 (16) | 5 (3) |
| **ART-experienced** | | | | | | | | | | |
| DTG | 5097 | 3338 (65) | 1 (0) | 1 (0) | 1694 (33) | 3390 (67) | 1 (0) | 881 (17) | 843 (17) | 45 (1) |
| BIC | 1765 | 0 (0) | 0 (0) | 0 (0) | 1765 (100) | 0 (0) | 0 (0) | 0 (0) | 1765 (100) | 0 (0) |
| EVG | 3580 | 0 (0) | 0 (0) | 0 (0) | 3580 (100) | 0 (0) | 0 (0) | 1152 (32) | 2428 (68) | 0 (0) |
| RAL | 1486 | 310 (21) | 0 (0) | 2 (0) | 1057 (71) | 410 (28) | 4 (0) | 836 (56) | 261 (18) | 92 (6) |
| DRV | 1879 | 405 (22) | 0 (0) | 7 (0) | 1373 (73) | 476 (25) | 4 (0) | 1017 (54) | 392 (21) | 84 (4) |

# Supplemental Table 3. Missing values in baseline and outcome variables

| **n (%)** | **N** | | **Low-density**  **lipoprotein**  **at baseline** | **Viral load**  **at baseline** | **CD4 at**  **baseline** | **VACS**  **index 2.0**  **at baseline** | **Viral load**  **at 6-mo** | **Viral load**  **at 12-mo** | **CD4**  **at 6-mo** | **VACS**  **index 2.0**  **at 6-mo** |
| --- | --- | --- | --- | --- | --- | --- | --- | --- | --- | --- |
| **ART-naive** | |  |  |  |  |  |  |  |  |  |
| DTG | 703 | | 132 (19) | 180 (26) | 172 (24) | 69 (10) | 213 (30) | 243 (35) | 241 (34) | 195 (28) |
| BIC | 316 | | 53 (17) | 73 (23) | 67 (21) | 34 (11) | 84 (27) | 123 (39) | 109 (34) | 104 (33) |
| EVG | 579 | | 121 (21) | 145 (25) | 144 (25) | 41 (7) | 157 (27) | 211 (36) | 177 (31) | 138 (24) |
| RAL | 121 | | 22 (18) | 39 (32) | 43 (36) | 14 (12) | 47 (39) | 59 (49) | 47 (39) | 38 (31) |
| DRV | 176 | | 45 (26) | 54 (31) | 57 (32) | 19 (11) | 58 (33) | 84 (48) | 61 (35) | 53 (30) |
| **ART-experienced** | |  |  |  |  |  |  |  |  |  |
| DTG | 5097 | | 778 (15) | 344 (7) | 743 (15) | 204 (4) | 994 (20) | 1324 (26) | 1434 (28) | 760 (15) |
| BIC | 1765 | | 270 (15) | 171 (10) | 333 (19) | 369 (21) | 436 (25) | 552 (31) | 634 (36) | 694 (39) |
| EVG | 3580 | | 580 (16) | 308 (9) | 504 (14) | 132 (4) | 740 (21) | 990 (28) | 920 (26) | 533 (15) |
| RAL | 1486 | | 232 (16) | 181 (12) | 216 (15) | 60 (4) | 354 (24) | 558 (38) | 421 (28) | 271 (18) |
| DRV | 1879 | | 306 (16) | 216 (11) | 297 (16) | 105 (6) | 448 (24) | 652 (35) | 543 (29) | 346 (18) |

# Supplemental Table 4. Deaths within 6 and 12 months

| **n (%)** | **N** | | **Deaths within 6 months** | **Deaths within 12 months** |
| --- | --- | --- | --- | --- |
| **ART-naive** | |  |  |  |
| DTG | 703 | | 24 (3.4) | 42 (6.0) |
| BIC | 316 | | 9 (2.8) | 16 (5.1) |
| EVG | 579 | | 12 (2.1) | 22 (3.8) |
| RAL | 121 | | 4 (3.3) | 8 (6.6) |
| DRV | 176 | | 5 (2.8) | 14 (8.0) |
| **ART-experienced** | |  |  |  |
| DTG | 5097 | | 129 (2.5) | 230 (4.5) |
| BIC | 1765 | | 25 (1.4) | 49 (2.8) |
| EVG | 3580 | | 48 (1.3) | 94 (2.6) |
| RAL | 1486 | | 52 (3.5) | 74 (5.0) |
| DRV | 1879 | | 50 (2.7) | 93 (4.9) |

# Supplemental Table 5. Initiation year of ART regimens

| **n (%)** | **DTG (N=5800)** | **BIC (N=2081)** | **EVG (N=4159)** | **RAL (N=1607)** | **DRV (N=2055)** |
| --- | --- | --- | --- | --- | --- |
| 2014 | 384 (7) | - | 475 (11) | 643 (40) | 755 (37) |
| 2015 | 1221 (21) | - | 522 (13) | 410 (26) | 433 (21) |
| 2016 | 1761 (30) | - | 1279 (31) | 263 (16) | 374 (18) |
| 2017 | 1334 (23) | - | 1138 (27) | 165 (10) | 271 (13) |
| 2018 | 767 (13) | 457 (22) | 568 (14) | 73 (5) | 135 (7) |
| 2019 | 284 (5) | 1374 (66) | 157 (4) | 50 (3) | 67 (3) |
| 2020 | 49 (1) | 250 (12) | 20 (0) | 3 (0) | 20 (1) |

# Supplemental Table 6. Exploration of reasons for discontinuing regimens

1. 6-month discontinuation

|  | **Regimen** | **6-month discontinuations^1^** | **Interruption, n (%)** | **Switch from multi-tablet regimen to single-tablet regimen, n (%)** | **Switch from boosted 4-drug regimen to 3- or 2-drug regimen, n (%)** | **Switch from 3-drug regimen to 2-drug regimen, n (%)** | **Discontinuation following virologic failure or non-response, n (%)** |
| --- | --- | --- | --- | --- | --- | --- | --- |
| **ART-naïve** |  |  |  |  |  |  |  |
|  | DTG | 31 | 9 (29) | 10 (32) | 0 (0) | 0 (0) | 1 (3) |
|  | BIC | 11 | 7 (64) | 0 (0) | 0 (0) | 0 (0) | 0 (0) |
|  | EVG | 30 | 3 (10) | 0 (0) | 26 (87) | 1 (3) | 1 (3) |
|  | RAL | 10 | 2 (20) | 4 (40) | 0 (0) | 0 (0) | 0 (0) |
|  | DRV | 24 | 4 (17) | 17 (71) | 17 (71) | 0 (0) | 0 (0) |
| **ART-experienced** |  |  |  |  |  |  |  |
|  | DTG | 193 | 37 (19) | 49 (25) | 5 (3) | 12 (6) | 3 (2) |
|  | BIC | 28 | 15 (54) | 0 (0) | 0 (0) | 2 (7) | 1 (4) |
|  | EVG | 125 | 12 (10) | 0 (0) | 91 (73) | 0 (0) | 3 (2) |
|  | RAL | 144 | 19 (13) | 45 (31) | 4 (3) | 3 (2) | 3 (2) |
|  | DRV | 161 | 18 (11) | 66 (41) | 74 (46) | 3 (2) | 11 (7) |

1. 12-month discontinuation

|  | **Regimen** | **12-month discontinuations^1^** | **Interruption, n (%)** | **Switch from multi-tablet regimen to single-tablet regimen, n (%)** | **Switch from boosted 4-drug regimen to 3- or 2-drug regimen, n (%)** | **Switch from 3-drug regimen to 2-drug regimen, n (%)** | **Discontinuation following virologic failure or non-response, n (%)** |
| --- | --- | --- | --- | --- | --- | --- | --- |
| **ART-naïve** |  |  |  |  |  |  |  |
|  | DTG | 53 | 12 (33) | 22 (42) | 0 (0) | 1 (2) | 2 (4) |
|  | BIC | 17 | 10 (59) | 0 (0) | 0 (0) | 0 (0) | 0 (0) |
|  | EVG | 57 | 9 (16) | 0 (0) | 43 (75) | 2 (4) | 2 (4) |
|  | RAL | 18 | 5 (28) | 7 (39) | 0 (0) | 0 (0) | 1 (6) |
|  | DRV | 31 | 3 (10) | 24 (77) | 24 (77) | 0 (0) | 1 (3) |
| **ART-experienced** |  |  |  |  |  |  |  |
|  | DTG | 344 | 51 (15) | 93 (27) | 8 (2) | 28 (8) | 15 (4) |
|  | BIC | 63 | 36 (57) | 0 (0) | 0 (0) | 7 (11) | 3 (5) |
|  | EVG | 260 | 24 (9) | 0 (0) | 194 (75) | 2 (1) | 11 (4) |
|  | RAL | 268 | 22 (8) | 106 (40) | 7 (3) | 7 (3) | 13 (5) |
|  | DRV | 286 | 38 (13) | 128 (45) | 129 (45) | 5 (2) | 21 (7) |

^1^ Among individuals with complete information on variables used in the outcome model.

# Supplemental Figure 1. Measuring windows


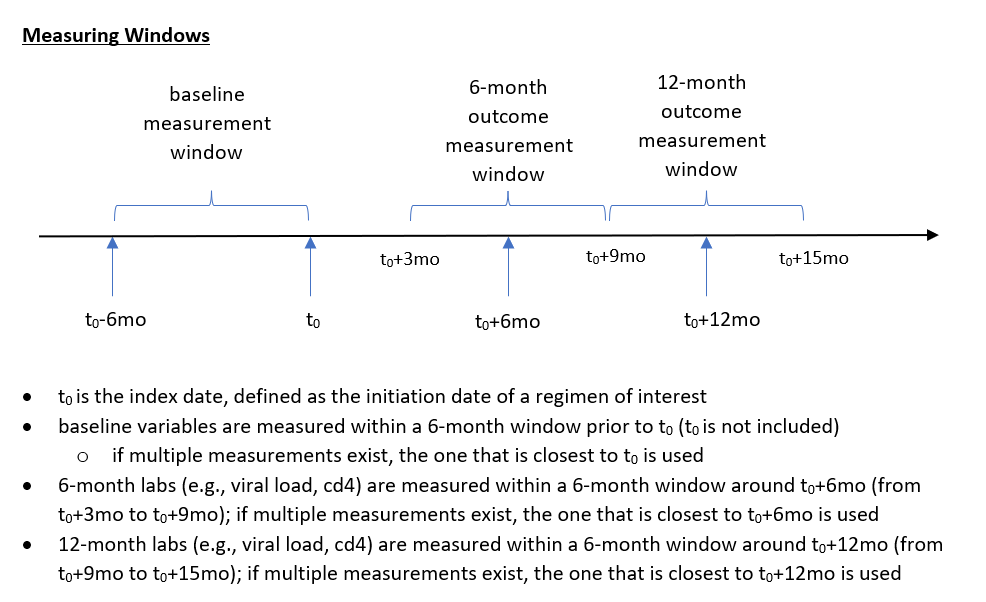


# Supplemental Figure 2. Effectiveness for those receiving BIC-, EVG-, RAL-, and DRV-based 3-drug regimens compared to those receiving DTG-based 3-drug regimen among ART-experienced PWH stratified by age group

1. Age<65


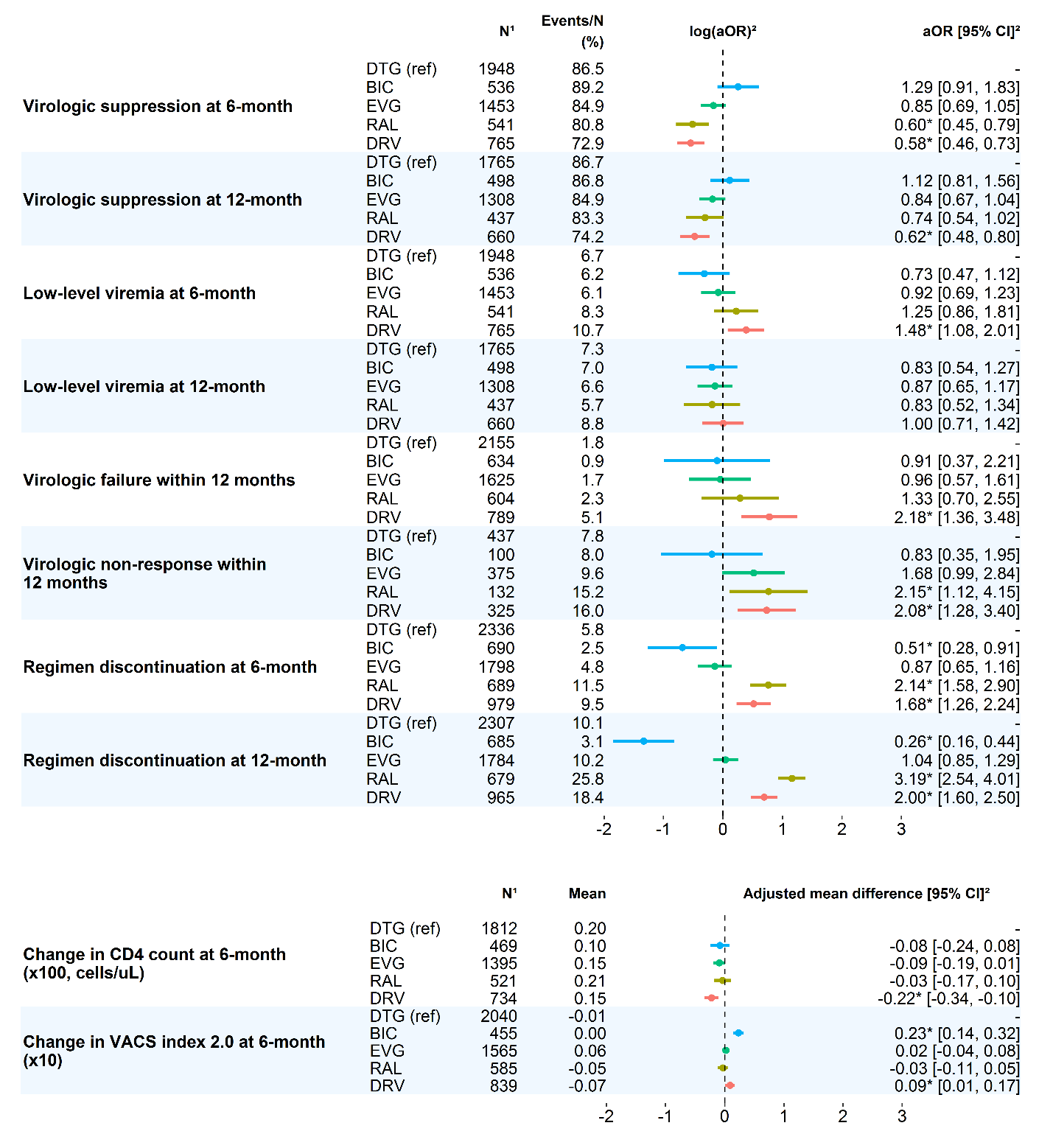


1. Age≥65


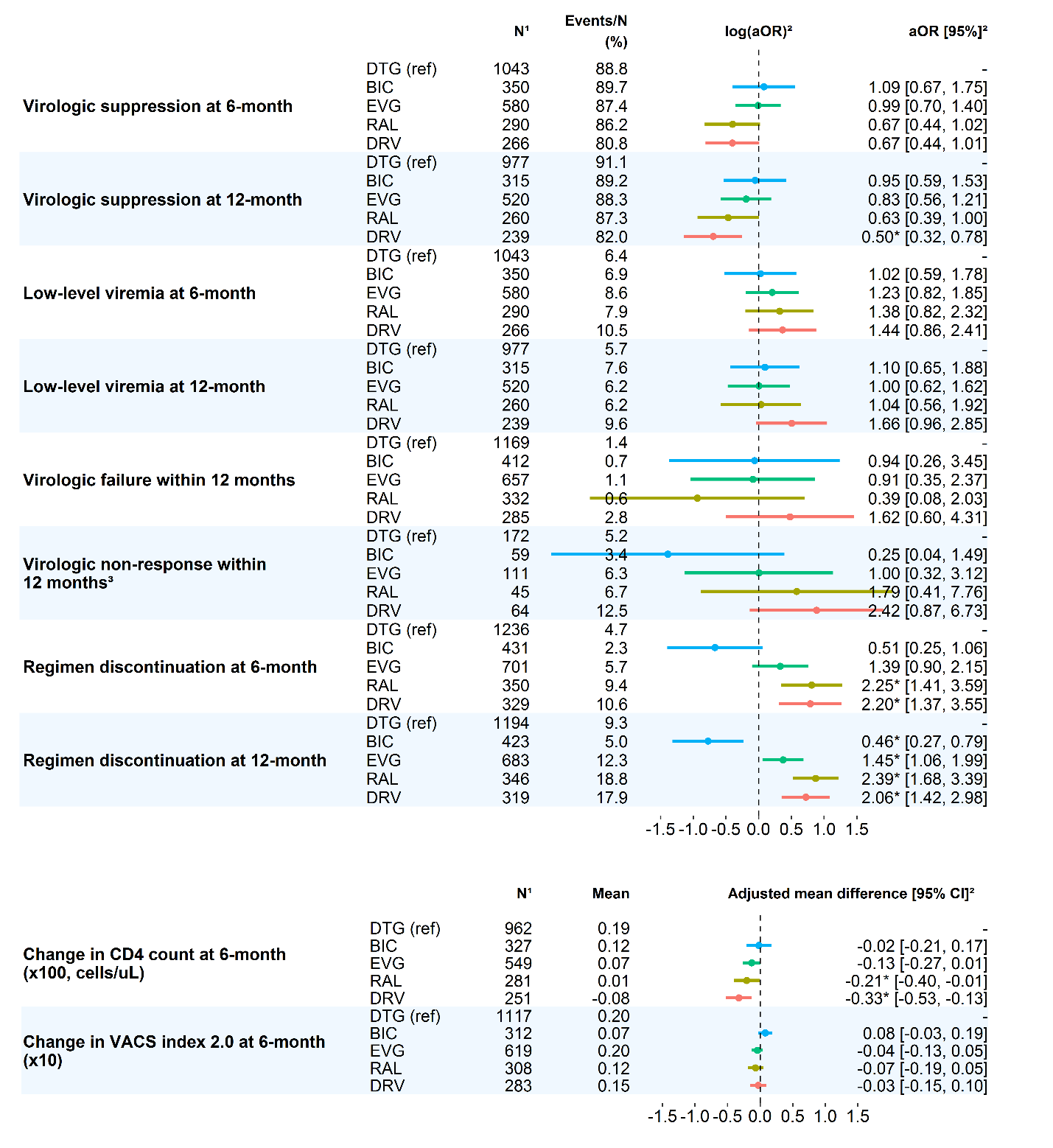


^1^ N represents the number of persons in each treatment group with complete information on variables used in the outcome model.

^2^ Estimates and confidence intervals were calculated from inverse-probability weighted models, adjusted for age, sex, race and/or ethnicity, region, smoking, alcohol use disorder, drug use and dependence, homelessness, baseline low-density lipoprotein, baseline CD4 count, baseline VL, baseline VACS 2.0 index, and years on ART regimen for ART-experienced.

^3^ Virologic non-response for ART-experienced was defined for individuals who were suppressed at baseline.

# Supplemental Figure 3. Effectiveness for those receiving BIC-, EVG-, RAL-, and DRV-based 3-drug regimens compared to those receiving DTG-based 3-drug regimen among ART-experienced PLWH stratified by hepatitis C virus (HCV)

1. No HCV


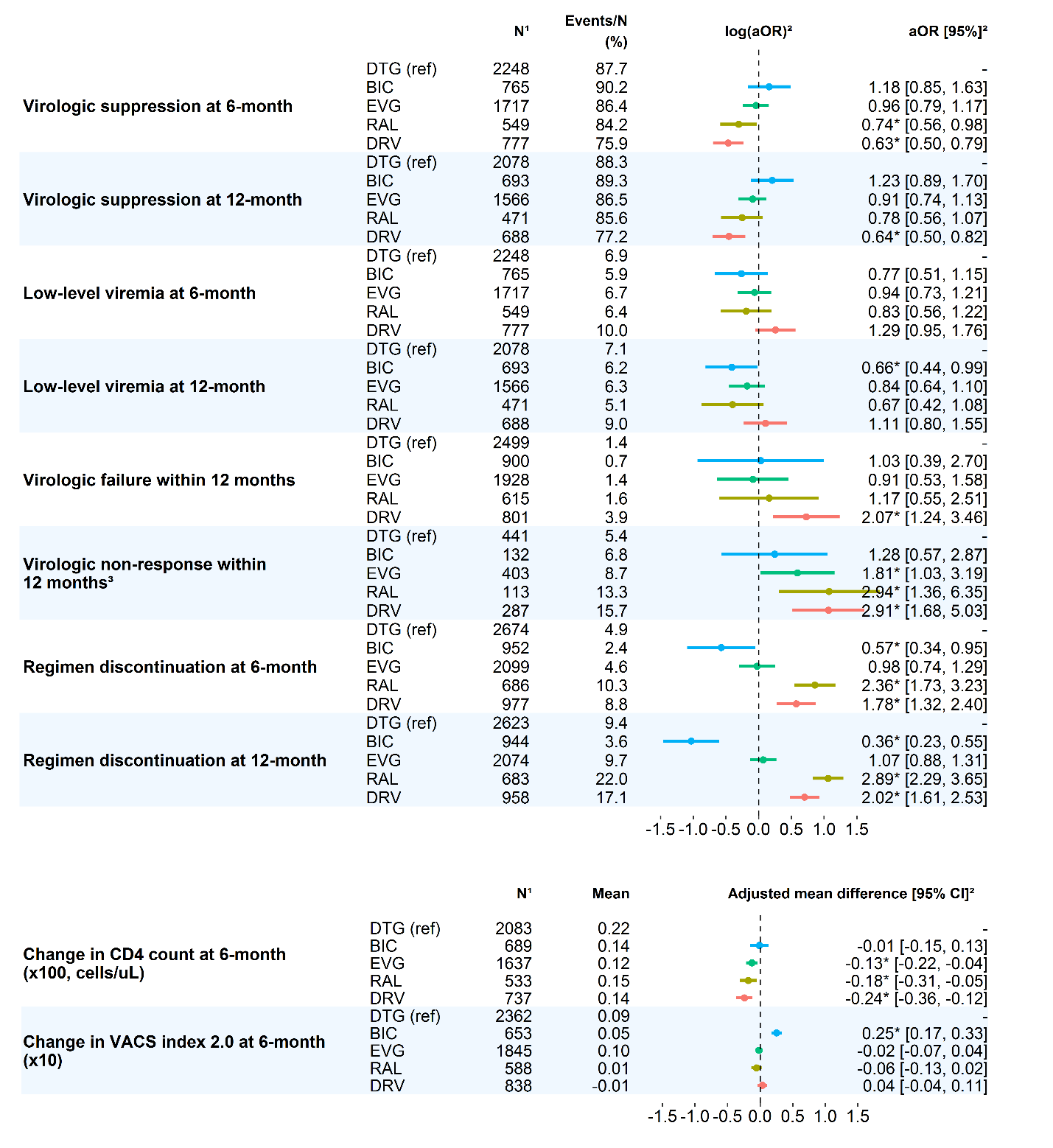


1. HCV


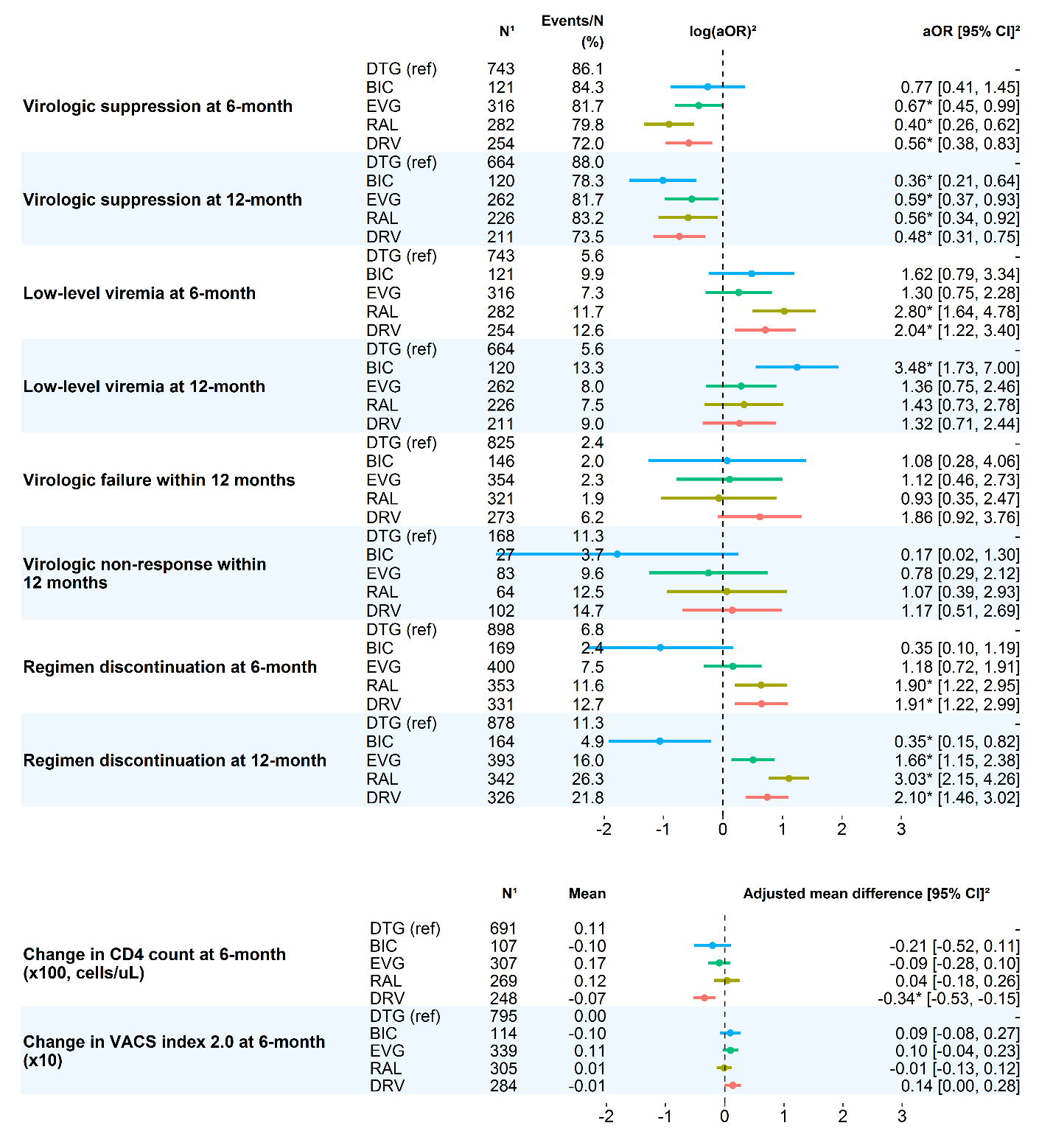


^1^ N represents the number of persons in each treatment group with complete information on variables used in the outcome model.

^2^ Estimates and confidence intervals were calculated from inverse-probability weighted models, adjusted for age, sex, race and/or ethnicity, region, smoking, alcohol use disorder, drug use and dependence, homelessness, baseline low-density lipoprotein, baseline CD4 count, baseline VL, baseline VACS 2.0 index, and years on ART regimen for ART-experienced.

^3^ Virologic non-response for ART-experienced was defined for individuals who were suppressed at baseline.

# Supplemental Figure 4. Comparison of treatment outcomes for those receiving BIC-, EVG-, RAL-, and DRV-based 3-drug regimens compared to those receiving DTG-based 3-drug regimen among A. ART-naïve and B. ART-experienced PWH where missing values were treated as a separate category in the weighting model

1. ART-naïve


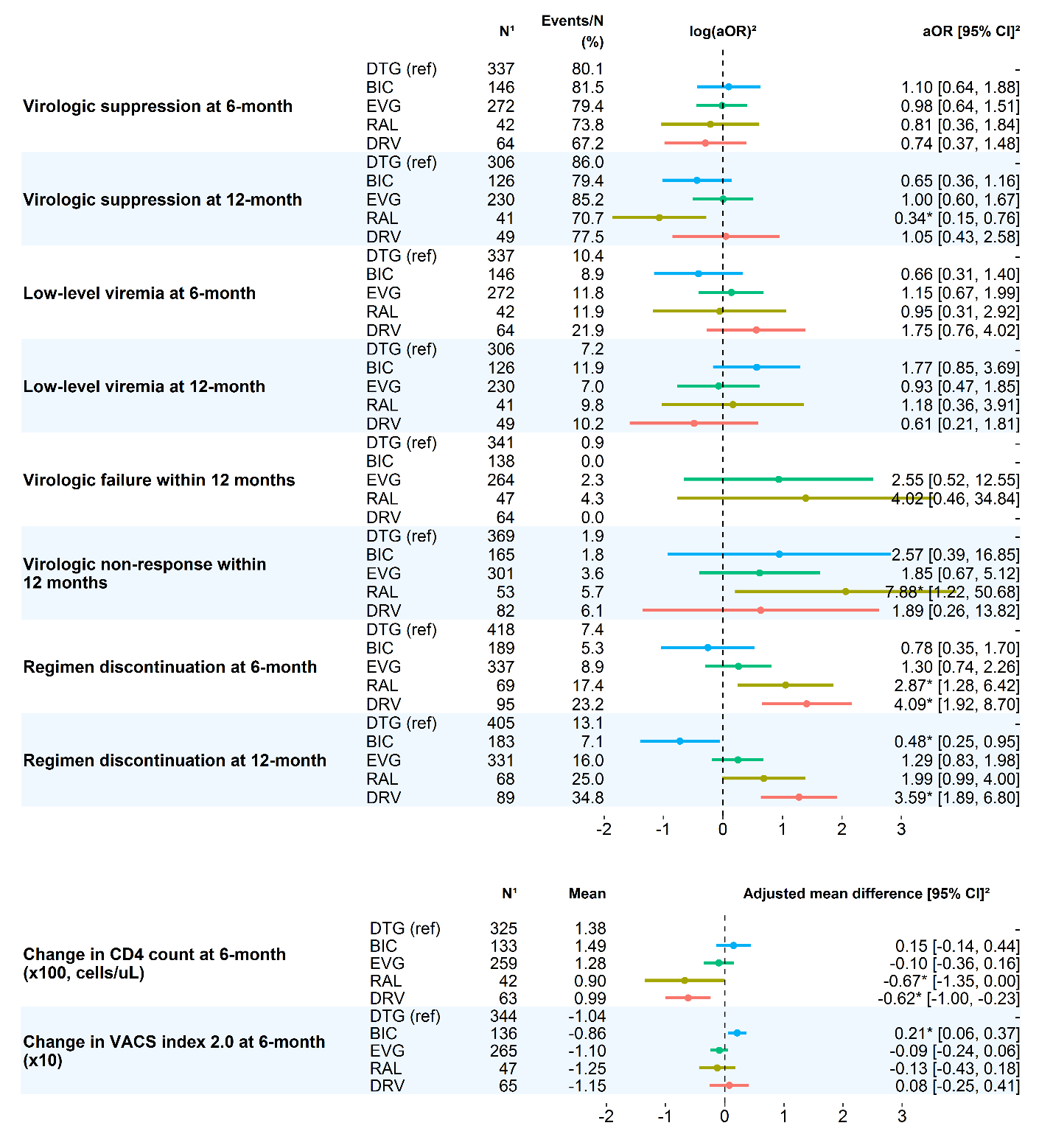


1. ART-experienced


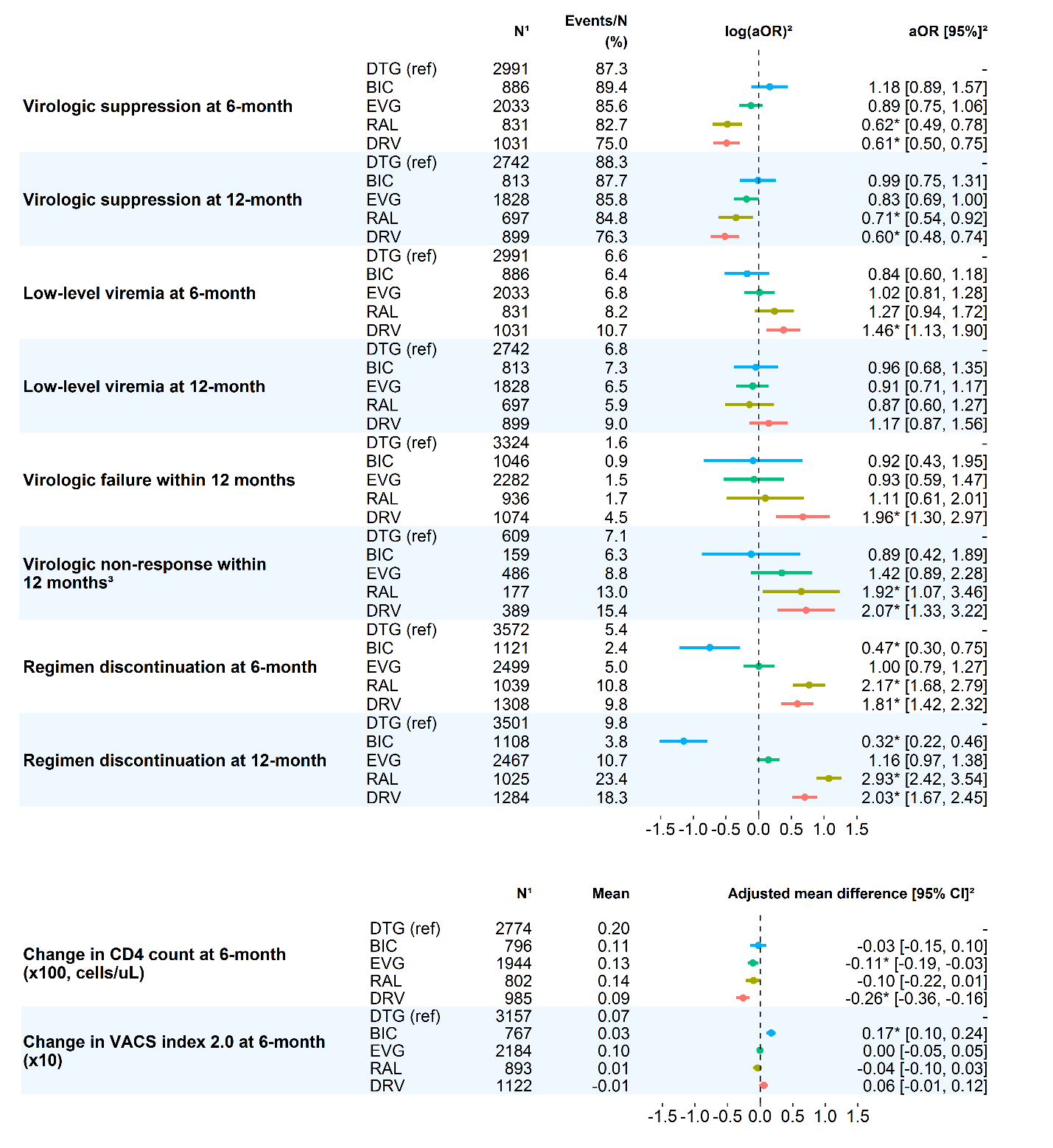


^1^ N represents the number of persons in each treatment group with complete information on variables used in the outcome model.

^2^ Estimates and confidence intervals were calculated from inverse-probability weighted models, adjusted for age, sex, race and/or ethnicity, region, smoking, alcohol use disorder, drug use and dependence, homelessness, baseline low-density lipoprotein, baseline CD4 count, baseline VL, baseline VACS 2.0 index, and years on ART regimen for ART-experienced.

^3^ Virologic non-response for ART-experienced was defined for individuals who were suppressed at baseline.

# Supplemental Figure 5. Comparison of methods

All outcomes were examined using crude analyses, multivariate models, multivariate models with inverse probability of treatment weighting (IPTW), and multivariate models with IPTW and inverse probability of censoring weighting (IPCW).

1. *Crude analyses*

Dichotomous outcomes were compared using logistic regression models and continuous outcomes were compared using linear regression models without adjusting for any baseline variables.

1. *Multivariate models*

Dichotomous outcomes were compared using logistic regression models and continuous outcomes were compared using linear regression models after adjusting for age, sex, race/ethnicity, region, smoking status, self-reported and ICD-9/10 based alcohol use disorder, ICD-9/10 based drug abuse and dependence, ICD-9/10 based homeless, level of low-density lipoprotein, years on ART medications (for ART-experienced), baseline CD4 cell count, baseline HIV-RNA level, and baseline VACS index 2.0.

1. *Multivariate models with IPTW*

Described in the manuscript.

1. *Multivariate models with IPTW and IPCW*

Missing outcomes may introduce selection bias. IPCW can be used to mitigate the potential selection bias due to missing outcomes by assuming that outcomes were missing randomly conditional on a set of variables. We estimated a logistic regression of whether an individual had an outcome on treatment and the same set of predictors used in the IPTW to obtain stabilized censoring weights, which were multiplied by stabilized treatment weights from IPTW to generate the final weights. The final weights were then trimmed at 99^th^ percentile and used to estimate the weighted outcome models.

Methods (2)-(4) generate similar point estimates and 95% confidence intervals. As an example, the figure below shows the comparison of virologic suppression at 6-month for those receiving BIC-, EVG-, RAL-, and DRV-based 3-drug regimens compared to those receiving DTG-based 3-drug regimen among ART-naïve and ART-experienced PLWH.


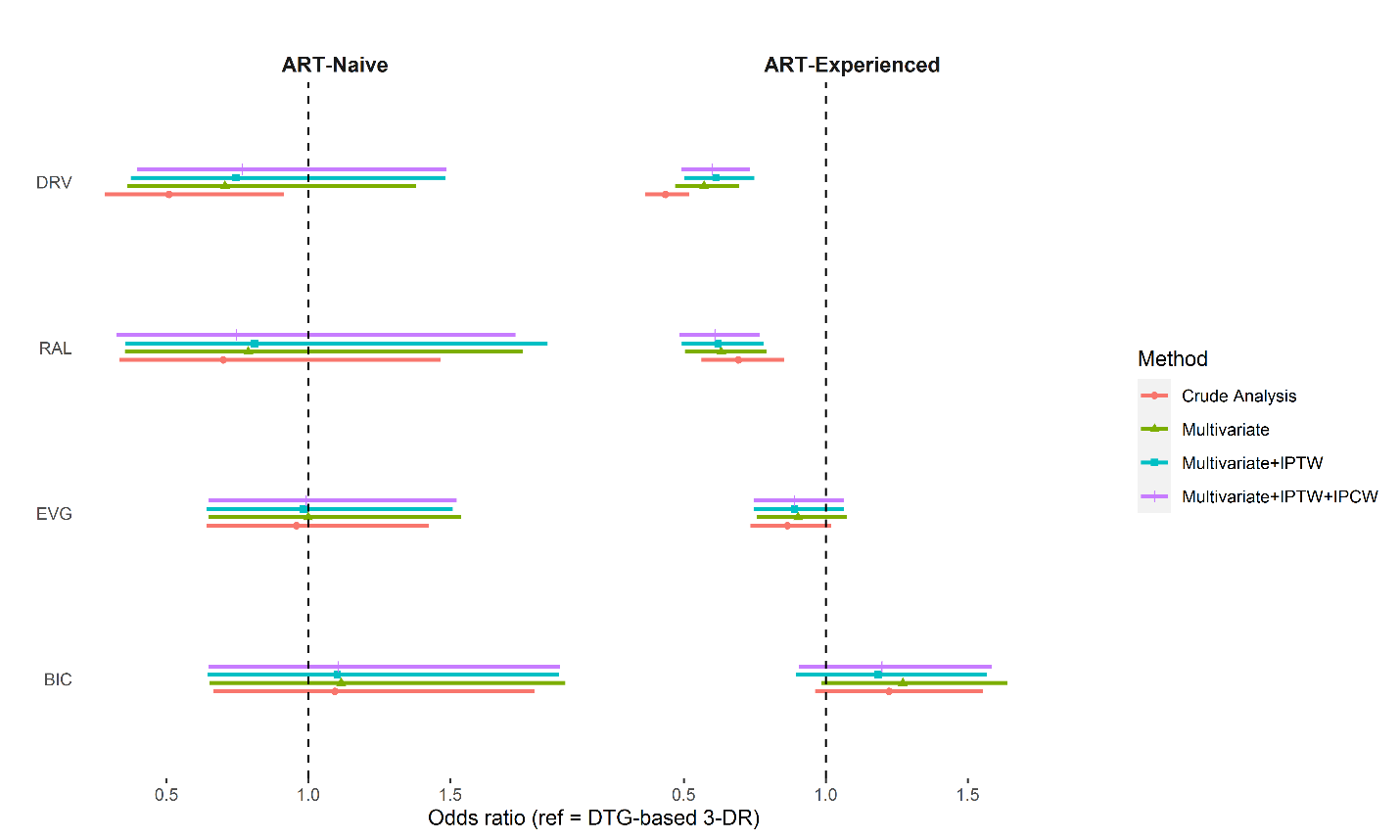


# Supplemental Figure 6. Comparison of treatment outcomes for those receiving BIC-based 3-drug regimens compared to those receiving DTG-based 3-drug regimen among A. ART-naïve and B. ART-experienced PWH during the period after the approval of BIC

1. ART-naïve


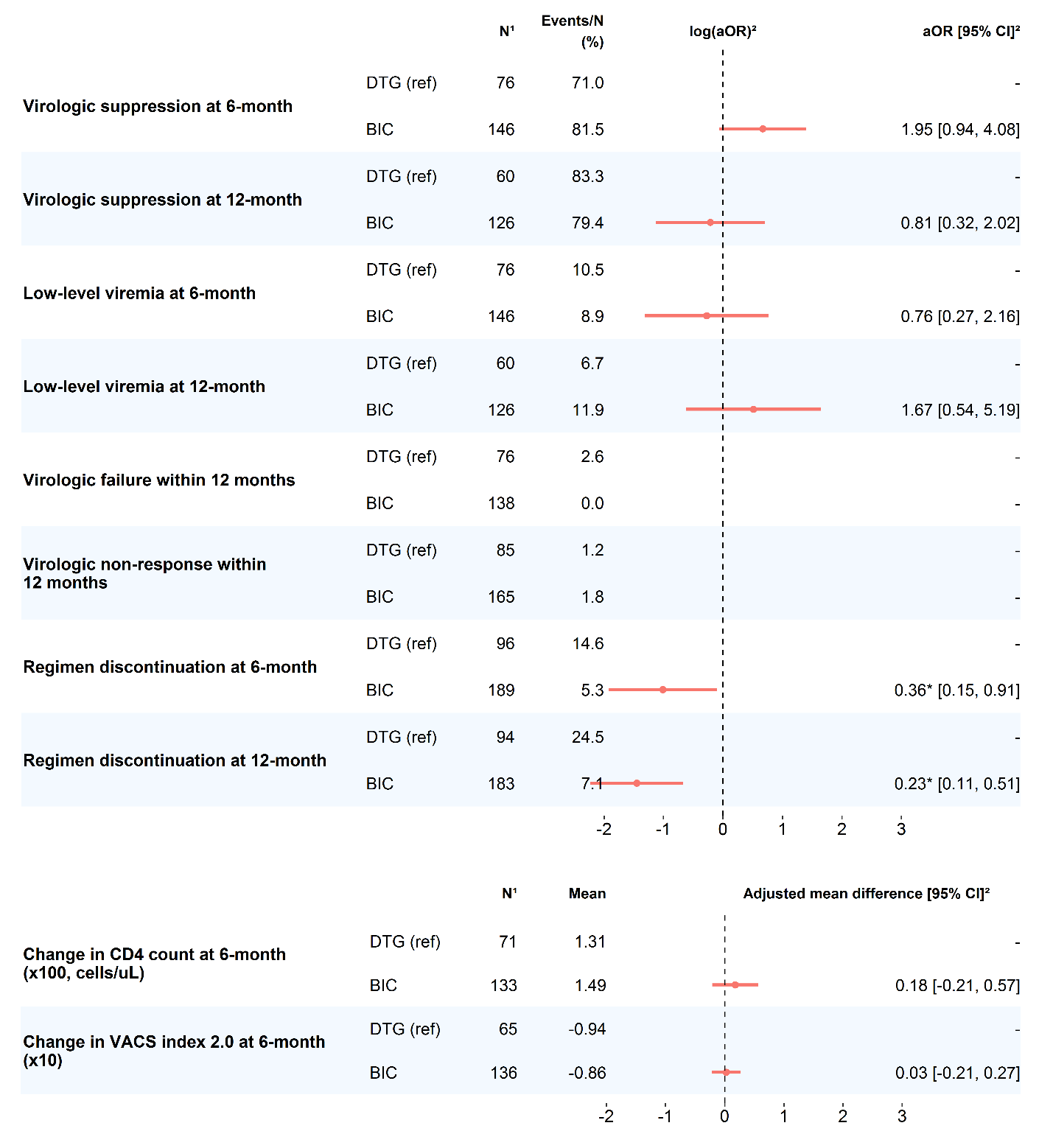


1. ART-experienced


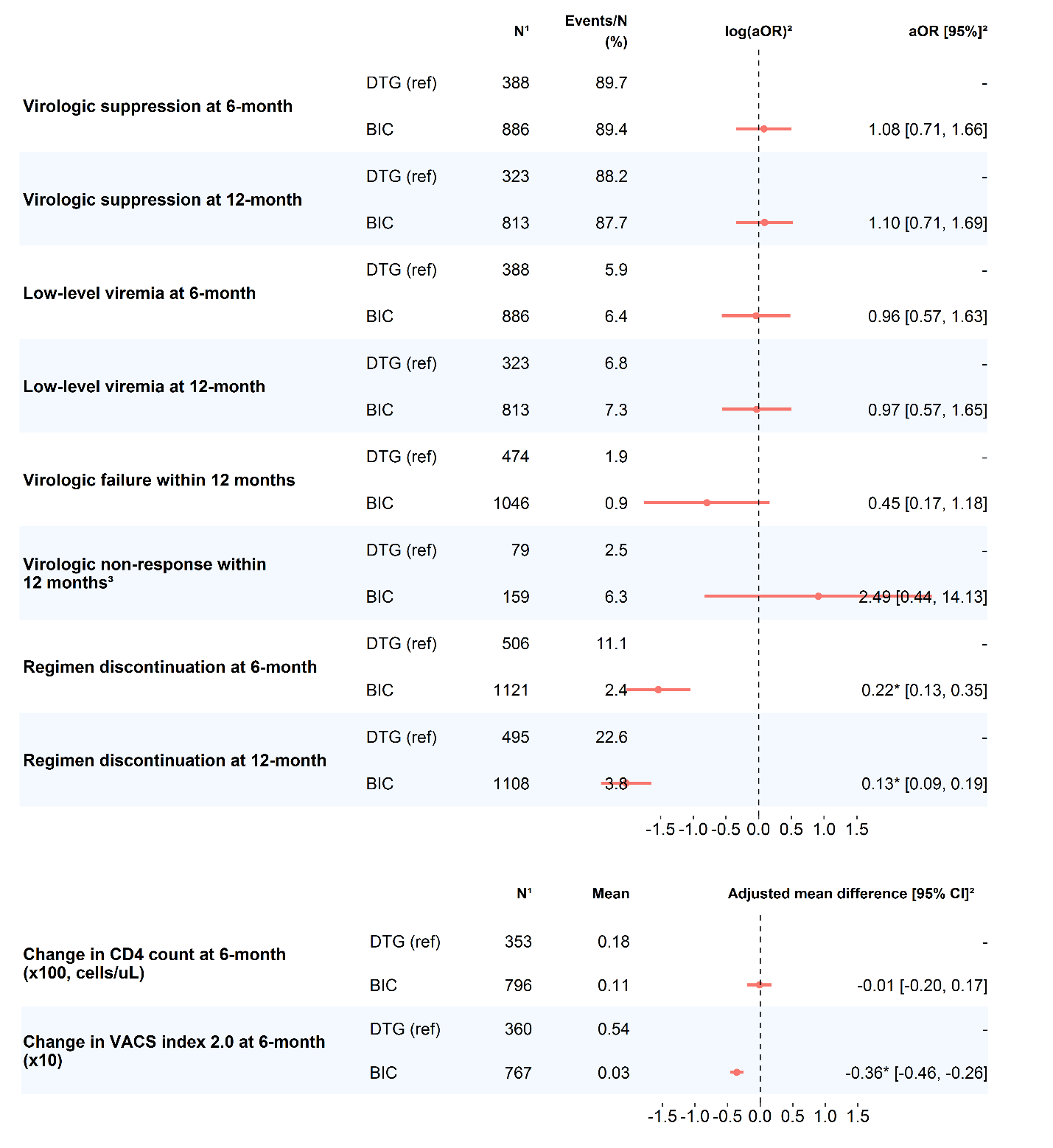


^1^ N represents the number of persons in each treatment group with complete information on variables used in the outcome model.

^2^ Estimates and confidence intervals were calculated from inverse-probability weighted models, adjusted for age, sex, race and/or ethnicity, region, smoking, alcohol use disorder, drug use and dependence, homelessness, baseline low-density lipoprotein, baseline CD4 count, baseline VL, baseline VACS 2.0 index, and years on ART regimen for ART-experienced.

^3^ Virologic non-response for ART-experienced was defined for individuals who were suppressed at baseline.
